# Race and Ethnicity and Clinician Linguistic Expressions of Doubt in Hospital Admission Notes

**DOI:** 10.1001/jamanetworkopen.2024.38550

**Published:** 2024-10-14

**Authors:** Courtney R. Lee, Jaya Aysola, Xinwei Chen, Eden Addisu, Ari Klein, Davy Weissenbacher, Graciela Gonzalez-Hernandez, Gary E. Weissman

**Affiliations:** 1 Leonard Davis Institute of Health Economics, University of Pennsylvania, Philadelphia; 2 Division of General Internal Medicine, Department of Medicine, University of Pennsylvania Perelman School of Medicine, Philadelphia; 3 Penn Medicine Center for Health Equity Advancement, Penn Medicine, Philadelphia, Pennsylvania; 4 Department of Biostatistics, Epidemiology, and Informatics, University of Pennsylvania, Philadelphia; 5 Department of Computational Biomedicine, Cedars-Sinai Medical Center, Los Angeles, California; 6 Division of Pulmonary, Allergy, and Critical Care Medicine, Department of Medicine, University of Pennsylvania, Philadelphia; 7 Palliative and Advanced Illness Research Center, University of Pennsylvania, Philadelphia, Pennsylvania

## Abstract

**Importance:**

Stigmatizing language in electronic health records (EHRs) can generate or reinforce negative stereotypes about patients from minoritized groups and erode their trust and experience. However, less is known about the use of such language to cast doubt on patient clinical history in hospital settings.

**Objective:**

To compare the prevalence of language expressing doubt about patient clinical history (eg, experiences and symptoms) in hospital admission notes by patient race and ethnicity.

**Design, Setting, and Participants:**

In this cohort study, natural language processing tools were applied to analyze 54 936 admission notes from 1249 clinicians about patients aged 18 years or older at admission and hospitalized at an academic health system in the Northeast US between January 1, 2018, and February 28, 2023. Data were analyzed from September 1, 2022, to July 31, 2023.

**Exposure:**

Patient race and ethnicity (non-Hispanic Black, non-Hispanic White, and racial and ethnic minoritized groups excluding non-Hispanic Black [includes multiple racial and ethnic groups, such as Hispanic and Asian]) as recorded in the EHR based on self-report or registrar determination.

**Main Outcome and Measure:**

Binary indicator for at least 1 term casting doubt on patient clinical history was defined using epistemic stance, a linguistic construct expressing a writer’s degree of certainty in information. Terms were manually validated via iterative review of notes by the study team.

**Results:**

Among 56 325 admission notes (mean [SD] age of patients, 55.9 [19.0] years; 30 913 notes among female patients [54.9%]; 25 649 notes among non-Hispanic Black patients [45.5%], 26 442 notes among non-Hispanic White patients [46.9%], and 2985 notes among members of racial and ethnic minoritized groups excluding non-Hispanic Black patients [5.3%]), we analyzed 54 936 admission notes that had no missing data. Among all analyzed admission notes, 39 023 notes (71.0%) contained doubt language. Notes written about non-Hispanic Black patients had increased odds of containing at least 1 word or phrase of doubt (adjusted odds ratio, 1.21; 95% CI, 1.14-1.28; *P* < .001) compared with notes among non-Hispanic White patients. Compared with notes among non-Hispanic White patients, notes written about members of racial and ethnic minoritized groups excluding non-Hispanic Black patients had similar odds of containing at least 1 term of doubt.

**Conclusion and Relevance:**

In this study, language casting doubt on patient clinical history was more likely to be found in notes of non-Hispanic Black patients. These results suggest that with the implementation of policies allowing patients full access to their health records, examining clinical documentation standards may be associated with improved patient experience.

## Key Points

**Question:**

Does the prevalence of language expressing doubt about patient clinical history in hospital admission notes vary by patient race and ethnicity?

**Findings:**

In this cohort study analyzing 54 936 admission notes for hospitalized adults, 71.0% of notes contained language casting doubt on patient clinical history. Notes of non-Hispanic Black patients were significantly more likely to contain such language compared with notes of non-Hispanic White patients.

**Meaning:**

These findings suggest that addressing these disparities in clinical documentation may be associated with improved patient trust and experience, especially with policies providing patients full access to health records.

## Teaser

This cohort study investigates the association of patient race and ethnicity with clinician expressions of doubt in hospital admission notes.

## Introduction

Racially and ethnically minoritized populations face disparities in health outcomes.^1,2^ While many factors contribute to these inequities, stigma plays an important role in shaping unequal care.^3,4,5^ Stigma uses labels to categorize individuals into social groups (eg, race and gender identity) and implicitly or explicitly associates them with negative attributes. After these attributes are invoked, the label can be used to rationalize status loss, discrimination, and the maintenance of social power structures.^6,7^ Patients from racially and ethnically minoritized groups frequently report experiencing stigma while seeking care through perceived discrimination or unfair judgment.^8,9^ Increasing evidence suggests that language in the electronic health record (EHR) can also generate or reinforce negative stereotypes that stigmatize patients from marginalized groups.^10,11,12^

Clinicians use stigmatizing language to negatively characterize patients, and recent work suggests that such language is more likely to be found in clinical notes of non-Hispanic Black patients.^10,11,12^ For example, clinicians are more likely to use negative descriptors (eg, aggressive or noncompliant) and document race in the health records of non-Hispanic Black patients compared with those of non-Hispanic White patients.^11,12,13^ While these recent studies used large-scale analyses to examine the use of negative descriptors, no large-scale study to our knowledge has investigated the role language may play in undermining the credibility of patients. Prior evidence shows that in ambulatory settings, clinicians were more likely to use language expressing doubt about non-Hispanic Black patients’ credibility.^10^ However, whether these same language patterns exist in hospital settings remains unknown.

High cognitive load, patient volume, and limited time in hospital settings may be associated with substantially different documentation patterns that rely more on heuristics or stereotypes.^14,15,16^ Brief, one-off encounters may or may not activate biases or stereotypes similar to those found in long-standing patient relationships.^17,18^ Additionally, admission notes can inform subsequent clinician-patient encounters and are essential to hospital staff communication and medical decision-making.^19^ Therefore, doubt language in these notes has the potential to bias perceptions of subsequent readers and undermine patient experience. Taken together, these facts suggest that hospital settings possess unique characteristics that may make them more susceptible to bias.

Understanding doubt language patterns in hospital settings may address the potential association of stigmatizing language with differences in hospital care. In this study, we used natural language processing techniques and manual validation to assess the prevalence of doubt language in admission notes at an academic health system and evaluate whether patient race and ethnicity were associated with use of such language.

## Methods

This cohort study was considered exempt from review and informed consent by the Institutional Review Board of the University of Pennsylvania because the data were deindentified as authorized by 45 CFR §46.104, category 4. This study follows the Strengthening the Reporting of Observational Studies in Epidemiology (STROBE) reporting guideline.

### Data Sources and Collection

We conducted a retrospective cohort study using EHR data from a large, urban academic health system in the Northeast United States that included 3 hospitals. Patients aged 18 years or older at admission and hospitalized between January 1, 2018, and February 28, 2023, were included in analyses. From the EHR data warehouse, we extracted patient demographics, diagnostic codes to calculate Elixhauser comorbidity index, and encounter characteristics. We also extracted admission note text and obtained a unique, deidentified indicator for the clinician who signed each note. For encounters that contained more than 1 admission note, we selected the first signed admission note for that encounter. We excluded observations that had missing data for any patient demographics (sex, race and ethnicity, marital status, and primary language) from analyses.

We used the Medical Information Mart for Intensive Care III (MIMIC-III) database version 1.4^20^ to identify and validate terms that represented doubt language. MIMIC-III contains deidentified admission notes from patients treated in intensive care units (ICUs) at Beth Israel Deaconess Medical Center in Boston, Massachusetts, between June 1, 2001, and October 31, 2012. These data were practical for annotation, allowing text sharing and review among team members. While templated text was present, this was small relative to the amount of free text based on manual review and included in analyses.

### Manual Validation of Doubt Language

We used the linguistic construct epistemic stance to measure language expressing doubt in admission notes. Epistemic stance refers to language expressing a writer’s perceived doubt or certainty about a statement^21^ and includes 2 types: evidentiality and epistemic modalities (Table 1).^22^ Evidentiality depicts the source or nature of the statement’s information.^23^ For example, a clinician may state, “the patient *reports* numbness for 10 hours” (neutral alternative: the patient had numbness for 10 hours). While the statement may not intentionally express doubt, it allows the clinician to present patient information and remain equivocal about the credibility of those assertions. Epistemic modality conveys the degree of certainty in the information’s credibility.^24^ In the statement, “the patient *claims* she took her insulin” (neutral alternative: the patient took her insulin), the clinician uses “claims” to convey a low degree of certainty in the patient’s insulin adherence.

**Table 1.** Examples of Doubt Language Documented in Admission Notes^a^

Doubt language type	Definition	Terms	Examples^b^	More neutral language
Epistemic modality	Validity of information is not assumed, but the level of certainty depends on the assertive word before or within the clause.	Claims, believes	She claims her last drink was 3 weeks ago. Patient does not believe that hepatitis C is active.	Her last drink was 3 weeks ago. Patient does not have active hepatitis.
Evidential	Indicates information was not personally obtained or observed by the writer but that information was obtained through a secondhand source.	Complains, denies, endorses, notes, reports, says, states, describes, mentions, per patient, according to	She reports taking all her medications. Patient states he does not drink regularly but endorses binging. Per patient she has a history of atrial fibrillation though not recorded.	She took all her medications. Patient does not drink regularly but she does binge drink. She has a history of atrial fibrillation though not recorded.

^a^ Doubt language refers to words or phrases that cast uncertainty upon a patient’s reported clinical history (eg, symptoms, experiences, or health behaviors). The linguistic construct epistemic stance was used to measure and capture doubt language. Epistemic stance refers to how writers use language to document their assessment of certainty in information (epistemic modality) and describe its source (evidential).

^b^ Representative examples are adapted from admission notes found in Medical Information Mart for Intensive Care III data.

To identify terms reflecting epistemic stance, we compiled a list of terms based on expert opinion and literature on epistemic stance and computational linguistics. We identified 62 relevant terms. We used regular expressions, a natural language processing exact string–matching technique, to capture these terms in MIMIC-III, accounting for verb tense variation (eg, denies, denied, and denying). We detected 33 terms. To ensure contextual accuracy (Table 1), we abstracted 25 random text samples per term and 3 team members (C.R.L., E.A., and A.K.) independently coded whether the term represented epistemic stance using a priori guidelines (eFigure 1 in Supplement 1). For instances where coders disagreed, a consensus decision was determined by group review.

Terms were included if at least 80% of samples were coded as epistemic stance. For terms below this threshold, we revised regular expressions and reannotated samples (eTable 1 in Supplement 1). Terms were eliminated if they did not meet the 80% accuracy rate or had fewer than 10 matches (eTable 2 in Supplement 1). We included 13 terms in the final analysis (Table 1), with an intercoder reliability of 0.78 and agreement between 86.9% and 92.5%. Further information about the validation process is reported in eFigures 2 and 3 and eTable 2 in Supplement 1.

### Outcome Ascertainment Through Automated Note Classification

The primary outcome was the binary presence or absence of at least 1 term conveying doubt about a patient clinical history (eg, symptoms, experiences, or health behaviors) at the level of each note. In sensitivity analyses described subsequently, we modeled the outcome as a count variable to reflect the rate of occurrences of doubt language in a note.

### Exposure Variable

The primary exposure was race and ethnicity as collected in the EHR based on self-report or registrar determination and combined in the same database item. Patients identifying as Black or African American were grouped as non-Hispanic Black, and those identifying as White were grouped as non-Hispanic White. Due to small prevalence, patients identifying as Alaskan Native, American Indian, Asian, Hispanic, Middle Eastern, Native Hawaiian or Pacific Islander, Portuguese, or multiracial or multiethnic (asked separately such that patients could select it as a separate category) were categorized as members of racial and ethnic minoritized groups (excludes non-Hispanic Black patients). We selected these categories because increased susceptibility to bias and stigmatizing language have been well documented in these groups.^10,11,12,15,24^ Race responses were recorded regardless of whether an individual answered yes to being Hispanic.

### Statistical Analysis

We reported summary statistics, including mean (SD) for continuous variables and counts and percentages for categorical variables. To evaluate the association between race and ethnicity and the presence of doubt language, we fit mixed-effects logistic regression models that included a random effect for clinicians and standard errors at the level of clinician to account for clustering of admission notes within clinicians and patterns in documentation. We included hospital fixed effects to account for hospital-level variation in documentation practices. Based on prior literature associating patient characteristics with stigmatizing language or clinicians’ implicit negative attitudes,^10,11,12,25,26^ models were adjusted for patient characteristics, including age, sex (male or female), primary language (English or not English), marital status (single or married), Elixhauser comorbidity index, insurance provider (employer based, Medicaid, or Medicare), discharge location (discharged to home, rehabilitation, or skilled nursing facility or self-discharge [ie, discharge against medical advice]), and whether the timing of the admission was before or after the start of the COVID-19 pandemic (encounter before vs on or after March 1, 2020).

#### Secondary Analyses

We also assessed the association between race and use of specific terms by fitting unadjusted models to each word or phrase for non-Hispanic Black and non-Hispanic White patients. We did not include correction for multiple comparisons given that models were unadjusted. We compared only these 2 groups given their high prevalence. Given prior findings of an interaction between race and sex in the association with doubt language,^10^ we repeated our main analyses with an interaction term for race and sex. Due to linguistic differences between evidential and epistemic modalities, we conducted post hoc secondary analyses by classifying terms into each category, and we repeated main analyses with each category as outcome. We also evaluated the association between race and ethnicity and the presence of any doubt language in a subgroup analysis of patients in the ICU.

#### Sensitivity Analyses

To evaluate whether findings were robust to variation in total note word count, we repeated analyses using a mixed-effects Poisson regression estimating how many times (reported as a rate ratio) each doubt word appeared relative to the total count of words. To determine whether associations between race and ethnicity and the presence of doubt terms were specific to doubt language rather than systematic differences in documentation practices, we conducted a falsification test using stop words (eg, *a* and *the*) that do not contribute meaning to a sentence on their own (eTable 3 in Supplement 1). We expected no differences in stop word use across patient race and ethnicity unless other systemic differences in clinical documentation existed. To evaluate this expectation, we refit the same mixed-effects Poisson regression as described previously, substituting the outcome with number of times a stop word occurred.

We used SAS statistical software version 9.4 (SAS Institute) to construct the data; we used R statistical software version 4.3.1 (R Project for Statistical Computing) and Stata statistical software version 16.1 (StataCorp) to conduct statistical analyses. We used a 2-sided *P* < .05 as the significance threshold. Analyses were performed between September 1, 2022, and July 31, 2023.

## Results

Among 56 325 admissions notes (mean [SD] age of patients, 55.9 [19.0] years; 30 913 notes among female patients [54.9%]; 25 649 notes among non-Hispanic Black patients [45.5%], 26 442 notes among non-Hispanic White patients [46.9%], and 2985 notes among members of racial and ethnic minoritized groups excluding non-Hispanic Black patients [5.3%]), we excluded 1389 hospital encounters (2.5%) that had missing data for at least 1 patient demographic during the study period (eFigure 4 in Supplement 1). We thus analyzed 54 936 admission notes from 1249 clinicians (median [IQR], 9 [3-21] notes per clinician) (Table 2). Terms conveying doubt appeared at least once in 39 023 analyzed admission notes (71.0%) (Table 3).

**Table 2.** Patient, Encounter, and Admission Note Characteristics

Characteristic	Admission notes, No. (%)			
	Hospitals A-C combined (N = 56 325)	Hospital A (n = 25 363)	Hospital B (n = 16 319)	Hospital C (n = 14 643)
Age, mean (SD), y	55.9 (19.0)	54.5 (18.6)	50.7 (19.7)	64.2 (15.7)
Sex				
Female	30 913 (54.9)	14 388 (56.7)	10 486 (64.3)	6039 (41.2)
Male	25 412 (45.1)	10 975 (43.3)	5833 (35.7)	8604 (58.8)
Race and ethnicity				
Asian or Pacific Islander	1430 (2.5)	683 (2.7)	589 (3.6)	158 (1.1)
Hispanic	210 (0.4)	69 (0.3)	100 (0.6)	41 (0.3)
Non-Hispanic Black	25 649 (45.5)	11 050 (43.6)	6202 (38.0)	8397 (57.3)
Non-Hispanic White	26 442 (46.9)	12 370 (48.8)	8550 (52.4)	5522 (37.7)
Other^a^	1345 (2.4)	684 (2.7)	335 (2.1)	326 (2.2)
Missing	1249 (2.2)	507 (2.0)	543 (3.3)	199 (1.4)
Marital status				
Married or life partner	26 474 (47.0)	12 451 (49.1)	8314 (50.9)	5709 (39.0)
Not married	29 727 (52.8)	12 852 (50.7)	7967 (48.8)	8908 (60.8)
Missing	124 (0.2)	60 (0.2)	38 (0.2)	26 (0.2)
Primary language				
English	55 063 (97.8)	24 836 (97.9)	15 841 (97.1)	14 386 (98.2)
Not English	1235 (2.2)	513 (2.0)	475 (2.9)	247 (1.7)
Missing	27 (<0.1)	14 (0.1)	3 (<0.1)	10 (0.1)
Insurance provider				
Medicaid	13 210 (23.5)	6384 (25.2)	3775 (23.1)	3051 (20.8)
Medicare	25 441 (45.2)	10 831 (42.7)	6036 (37.0)	8574 (58.6)
Employer based^b^	17 666 (31.4)	8143 (32.1)	6505 (39.9)	3018 (20.6)
Missing	8 (<0.1)	5 (<0.1)	3 (<0.1)	0 (<0.1)
Admission location				
Emergency department	35 031 (62.2)	15 079 (59.5)	8023 (49.2)	11 929 (81.5)
OBGYN or elective admission	21 288 (37.8)	10 284 (40.5)	8295 (50.8)	2709 (18.5)
Missing	6 (<0.1)	0 (<0.1)	1 (<0.1)	5 (<0.1)
Discharge location				
Home, rehabilitation, or skilled nursing facility	55 475 (98.5)	25 054 (98.8)	16 072 (98.5)	14 349 (98.0)
Self-discharge^c^	850 (1.5)	309 (1.2)	247 (1.5)	294 (2.0)
Encounter length, mean (SD), d	6.1 (9.4)	6.8 (11.3)	4.8 (6.5)	6.3 (8.3)
Elixhauser comorbidity index, mean (SD)	6.2 (8.9)	6.9 (9.4)	3.7 (7.6)	7.6 (8.9)
Timing of encounter^d^				
Before COVID-19 pandemic	24 912 (44.2)	11 157 (44.0)	7293 (44.7)	6462 (44.1)
During or after COVID-19 pandemic	31 413 (55.8)	14 206 (56.0)	9026 (55.3)	8181 (55.9)
No. of words per note, median (IQR)	1533 (996-2345)	1680 (1094-2574)	1337 (907-2010)	1527 (980-2362)
No. of notes per clinician, median (IQR)	9 (3-21)	5 (2-14)	4 (1-12)	4 (2-11)

Abbreviation: OBGYN, obstetrics and gynecology.

^a^ Other race and ethnicity groups include Alaskan Native, American Indian, Middle Eastern, Native Hawaiian, Portuguese, and multiracial or multiethnic.

^b^ Employer-based insurance provider includes employer-based, self-pay, and private insurance providers.

^c^ Self-discharge refers to a patient who discharges against medical advice.

^d^ We designated March 1, 2020, as the approximate date when clinical practice behaviors changed in response with the COVID-19 pandemic.

**Table 3.** Frequency of Doubt Language in Admission Notes

Keyword	Notes with ≥1 doubt language keyword, No. (%)^a^			
	Hospitals A-C combined (N = 54 936)	Hospital A (n = 24 783)	Hospital B (n = 15 744)	Hospital C (n = 14 409)
Any keyword	39 023 (71.03)	16 957 (68.42)	11 563 (73.44)	10 503 (72.89)
Complains	2968 (5.40)	1204 (4.86)	786 (4.99)	978 (6.79)
Denies	23 150 (42.10)	9402 (37.94)	7619 (48.39)	6129 (42.54)
Endorses	6507 (11.84)	3078 (12.42)	1729 (10.98)	1700 (11.80)
Notes	1938 (3.53)	974 (3.93)	390 (2.48)	574 (3.98)
Reports	23 047 (41.95)	10 818 (43.65)	6610 (41.98)	5619 (39.00)
Says	2283 (4.16)	973 (3.93)	661 (4.20)	649 (4.50)
States	11 412 (20.77)	4520 (18.24)	3366 (21.38)	3526 (24.47)
Claims	146 (0.27)	42 (0.17)	46 (0.29)	58 (0.40)
Describes	2535 (4.61)	995 (4.01)	756 (4.80)	784 (5.44)
Mentions	122 (0.22)	54 (0.22)	29 (0.18)	39 (0.27)
Believes	371 (0.68)	187 (0.75)	104 (0.66)	80 (0.56)
According	116 (0.21)	60 (0.24)	28 (0.18)	28 (0.19)
Per patient	4105 (7.47)	2120 (8.55)	1150 (7.30)	835 (5.79)

^a^ Doubt language refers to terms that cast uncertainty on a patient’s reported clinical history (eg, symptoms, experiences, or health behaviors). The linguistic construct epistemic stance was used to measure and capture doubt language. Epistemic stance refers to how writers use language to document their assessment of certainty in information (epistemic modality) and describe its source (evidential).

**Figure 1.**
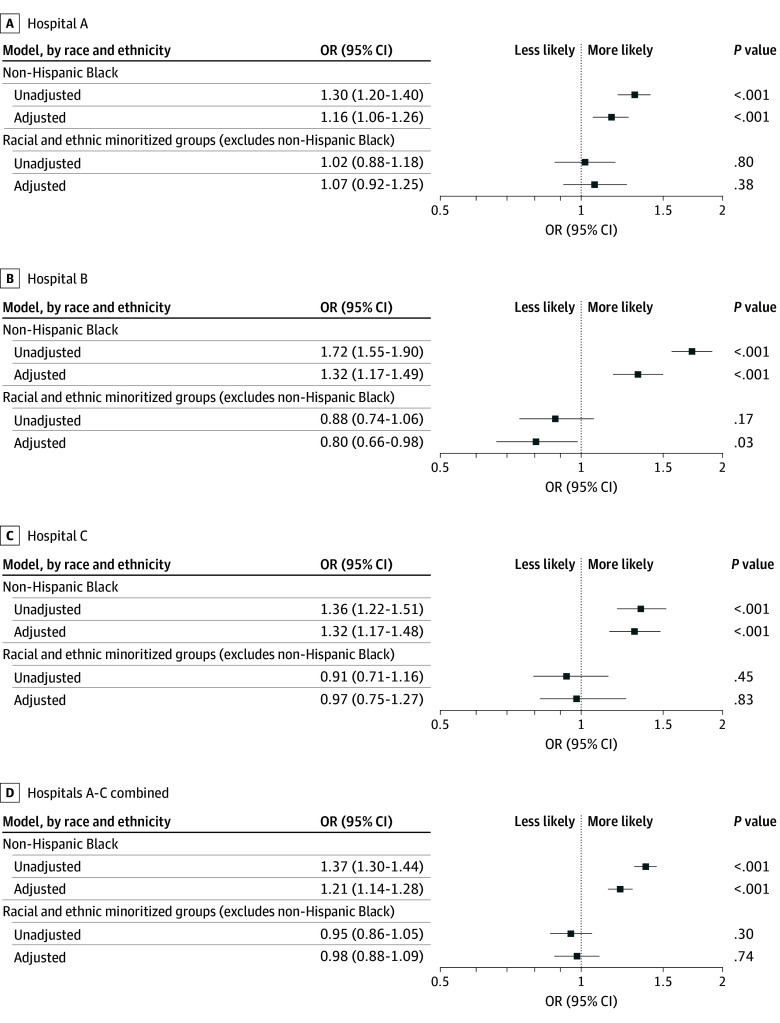
Unadjusted and Adjusted Associations Between Patient Race and Ethnicity and the Presence of Any Doubt Language Plots show odds of 1 or more occurrence of doubt language, which refers to words or phrases that cast uncertainty on a patient’s reported clinical history (eg, symptoms, experiences, or health behaviors). Examples include *claims*, *reports*, and *denies*. Comparisons are between non-Hispanic Black patients and non-Hispanic White patients and between racial and ethnic minoritized groups (excluding non-Hispanic Black and African American patients and including Alaskan Native, American Indian, Asian, Middle Eastern, Native Hawaiian or Pacific Islander, Portuguese, or multiracial or multiethnic patients) and non-Hispanic White patients. In all models a random effect was included for clinicians, standard errors were clustered at the level of the clinician to reflect associations in clinician documentation practices, and hospital fixed effects were included to account for hospital-level variation in documentation practices. Adjusted models included adjustment for patient (age, sex, primary language, marital status, Elixhauser comorbidity index, and insurance provider) and encounter (discharge location and timing of the hospital encounter before or after the start of the COVID-19 pandemic) characteristics. OR indicates odds ratio.

**Figure 2.**
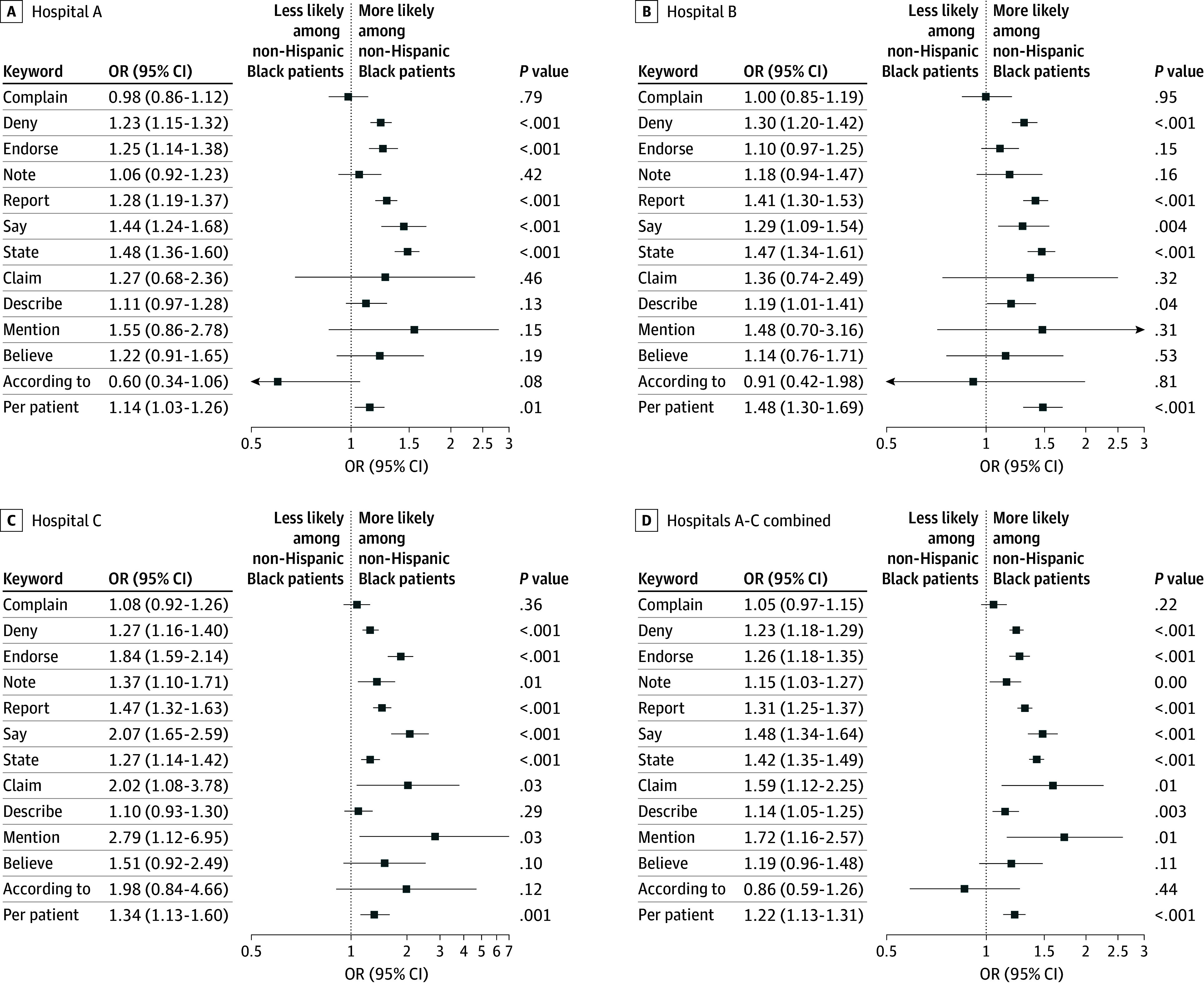
Unadjusted Odds Ratios of the Occurrence of Specific Doubt Language Plots show odds of the occurrence of specific doubt language, which are words or phrases that cast uncertainty upon a patient’s reported clinical history (eg, symptoms, experiences, or health behaviors). Examples include *claims*, *reports*, and *denies*. Comparisons are between non-Hispanic White patients and non-Hispanic Black patients only because of the relatively large prevalence of patients in these groups. In all models a random effect was included for clinicians, standard errors were clustered at the level of the clinician to reflect associations in clinician documentation practices, and hospital fixed effects were included to account for hospital-level variation in documentation practices. No models included adjustment for patient or encounter characteristics and thus did not include corrections for multiple comparisons. OR indicates odds ratio.

Among all hospitals, notes written about non-Hispanic Black patients had higher adjusted odds of containing doubt language (adjusted odds ratio [aOR], 1.21; 95% CI, 1.14-1.28; *P* < .001) compared with notes among non-Hispanic White patients (Figure 1). We observed similar patterns for each hospital, but the effect size varied by site. Additionally, we observed similar patterns for each word by site, but *claims*, *according to*, and *mentions* had effect sizes and significance that varied by hospital (Figure 2). We did not observe an interaction between patient sex and race and ethnicity and the presence of doubt language (eTable 4 in Supplement 1). Compared with notes among non-Hispanic White patients, notes of non-Hispanic Black patients had higher odds of containing an evidential modality (aOR, 1.41; 95% CI, 1.33-1.49; *P* < .001) and an epistemic modality (aOR, 1.27; 95% CI, 1.05-1.54; *P* = .02) (eTable 5 in Supplement 1). In a subgroup of patients in the ICU, notes written about non-Hispanic Black patients had higher adjusted odds of containing doubt language compared with those among non-Hispanic White patients (aOR, 1.22; 95% CI, 1.08-1.39; *P* = .002) (eTables 6-8 and eFigure 5 in Supplement 1).

These findings were robust to sensitivity analyses. We found consistent associations between race and ethnicity and the rate of doubt language occurrences when accounting for total word count (eTable 9 in Supplement 1). We observed no association between race and ethnicity and use of stop words (eTable 10 in Supplement 1).

## Discussion

In this cohort study, we analyzed 54 936 admission notes from 1249 clinicians at a large academic health system and found that most admission notes contained language expressing doubt about patient-reported clinical histories. In adjusted analyses, admission notes written about non-Hispanic Black patients were more likely to contain doubt language than those of non-Hispanic White patients. These findings have several important implications for measuring and addressing biases in clinical documentation.

Medicine, like other professions, has its own unofficial written language rooted in tradition. For nearly 200 years, words such as “denies” or “complains” have described patient symptoms in the medical literature.^27,28^ Trainees are acculturated to this written language through didactics, standardized patients, and role modeling from senior trainees or preceptors.^29,30,31^ Therefore, clinicians may not perceive these words as inherently disparaging and may use them more frequently to appear impartial. However, our findings suggest that although these words are common, they are used differentially between non-Hispanic Black and non-Hispanic White patients. Thus, these seemingly innocuous words may reflect systemic biases that can undermine racially and ethnically minoritized patients’ credibility.

Clinicians may be more likely to express credibility bias because, like the general population, they may unconsciously associate negative racial stereotypes with non-Hispanic Black patients.^25,32^ During interpersonal communication, credibility is assessed based on 3 factors: competence (perceived ability to communicate or interpret an experience), sincerity (perceived trustworthiness), and sociability (perceived likeability or warmth).^33^ During patient interactions, clinicians may use patient characteristics, such as race and ethnicity, to evaluate a patient’s credibility (or lack thereof) even if subconsciously.^34^ Biases or stereotypes associating non-Hispanic Black patients with being less cooperative,^35^ compliant,^36^ likable,^37^ or effective as communicators^38^ could adversely affect clinician perceptions of these patients’ credibility. Additionally, high levels of cognitive load,^14^ burnout,^39^ and patient intersectional identities with other stigmatized groups (eg, low socioeconomic status) or health conditions (eg, sickle cell anemia)^26^ may exacerbate biases that are associated with these negative perceptions.

Direct and indirect outcomes associated with doubt language constitute an understudied area. While doubt language may appear innocuous on its own, systematic biases in its use may have negative spillover associations with patient experience. For example, one study^40^ found that among 22 959 patients who reviewed their clinic notes, 10% reported feeling judged or offended by something they read. The presence of doubt language may exacerbate non-Hispanic Black patients’ existing feelings of mistrust and disrespect as being knowledgeable about their own symptoms.^41^ With the recent passage of the 21st Century Cures Act, health systems are now required to provide patients with full health record access. Thus, if examining clinician language is unaddressed, non-Hispanic Black patients may continue to experience less satisfaction with their care and be less likely to engage with health care in the future.^42,43^ Additionally, doubt language may negatively influence subsequent clinician attitudes or transmit biased documentation habits to trainees.^23^ These potential outcomes are particularly salient for admission notes given that they lay the foundation for decision-making and subsequent encounters.

Our findings contribute to the increasing body of literature on stigmatizing language in health records. First, our findings are consistent with prior work in the outpatient setting, which found that the notes of non-Hispanic Black patients had 1.25 times greater odds of containing doubt language than those of non-Hispanic White patients.^10^ Second, our results align with several previously reported analyses of admission notes that demonstrated that non-Hispanic Black patients had significantly greater odds of having negative descriptors in their notes compared with non-Hispanic White patients.^11,12^ To our knowledge, this study is the first large-scale analysis of admission notes to evaluate whether patient race and ethnicity were associated with doubt language in a hospital setting. Third, this study demonstrates that applying conventional linguistic constructs to medical settings may help us better understand and quantify stigmatizing language.

Many clinicians may find it challenging to document patient clinical history without using doubting words; therefore, such language may not necessarily reflect conscious or unconscious attitudes about patients. As a result, it is important to note that more work is needed to determine whether doubt language reflects the clinicians’ state of mind and whether such language conveys doubt to subsequent clinicians. While this study was not designed to make inferences about other sociodemographic attributes, these exploratory analyses suggest that further work is needed to understand the role that other patient attributes (eg, language or insurance type) may play in patient credibility.

### Limitations

This study has limitations. First, we used racial and ethnic categories identified from the EHR that may not accurately reflect patient self-identification. However, prior studies suggest that there is considerable concordance with patient self-report and EHR-documented race and ethnicity.^44^ Second, we conducted this study from admissions notes within 1 institution, which limits the generalizability of findings to other health systems given that language use may vary. Third, while we compiled a list of doubt language from existing medical and nonmedical literature, there is no consensus on doubt language in medical text. Although we developed an approach to validate the use of these words, we did not validate their use in the analytic sample. Therefore, it is possible that terms we captured in the notes of the analytic sample were not used in a stigmatizing manner. Conversely, we may have missed occurrences of doubt language in the primary cohort if not initially captured in MIMIC-III. Fourth, we used a manual validation process and an 80% threshold for accuracy to verify terms reflected uncertainty in patient clinical history. However, this does not ensure that every instance of a term produced the same findings, and this accuracy threshold may have introduced an error rate of up to 20% in the regular expression matching. It is unlikely that the error rate would differ by race and ethnicity as an alternative explanation for the study findings. However, future research efforts should integrate statistical natural language processing methods with additional layers of manual validation in analyzing linguistic constructs related to bias in large EHR datasets. Fifth, criteria used to define doubt language may have contributed to the estimated prevalence, although we were likely underestimating the total amount of doubt language in admission notes given that we excluded words that reflected doubt less than 80% of the time. Additionally, we did not include quotations, previously associated with doubt expressions, because they may suggest other forms of bias, such as highlighting irrational behavior or disapproval.^10^

## Conclusions

Findings from this cohort study suggest that language casting doubt on patient reports was highly prevalent in admission notes and more likely to be found in notes of non-Hispanic Black patients. Future efforts are needed to understand and address individual, interpersonal, and structural factors associated with this language and to quantify any downstream outcomes in patient experiences.

## Notes

Supplement 1. **eFigure 1.** Annotation Guide for Clinician Doubt in Admission Notes **eTable 1.** Extraction Details and Examples for Evidential and Epistemic Modality Terms **eTable 2.** Accuracy Rate and Percent Agreement for a Random Set of Text Samples for Each Term **eFigure 2.** Approach to Semantic Annotation of Epistemic Stance Terms in Health Records **eFigure 3.** Overall Percentage Agreement and Intercoder Reliability of Random Text Samples **eTable 3.** List of Stop Words Used in Regression Estimates of Falsification Test **eFigure 4.** Flow Diagram from Sample Population to Study Population **eTable 4.** Regression Estimates of Unadjusted and Adjusted Odds Ratios Between Patient Characteristics and the Presence of Any Doubt Language in Admission Notes, by Hospital Site **eTable 5.** Unadjusted Odds Ratios of Occurrence of Evidential and Epistemic Modality by Race **eTable 6.** Subgroup Analysis of Intensive Care Unit Documentation: Patient, Encounter, and Admission Note Characteristics by Hospital Site **eTable 7.** Subgroup Analysis of Intensive Care Unit (ICU) Documentation: Number of Times Doubt Language Occurred in ICU Admission Notes by Hospital Site **eTable 8.** Subgroup Analysis of Intensive Care Unit Documentation: Regression Estimates of Unadjusted and Adjusted Odds Ratios Between Patient Characteristics and the Presence of Any Doubt Language in Admission Notes by Hospital Site **eFigure 5.** Subgroup Analysis of Intensive Care Unit Documentation: Unadjusted and Adjusted Associations Patient Race and Ethnicity and the Presence of Doubt Language by Hospital Site **eTable 9.** Unadjusted and Adjusted Regression Estimates of Patient Characteristics and Occurrences of Doubt Language **eTable 10.** Falsification Test: Unadjusted and Adjusted Regression Estimates of Patient Characteristics and Rate Ratios of Stop Words

Supplement 2.
Data Sharing Statement

## References

[1] Centers for Disease Control and Prevention. CDC Health Disparities and Inequalities Report—United States, 2013. MMWR Morb Mortal Wkly Rep. 2013;62(3):1-189. 24264482

[2] Meyers D, Brady J, Grace E, . 2021 National Healthcare Quality and Disparities Report. Agency for Healthcare Research and Quality; 2021. Accessed September 6, 2024. https://www.ncbi.nlm.nih.gov/books/NBK578529/pdf/Bookshelf_NBK578529.pdf

[3] Green CR, Anderson KO, Baker TA, . The unequal burden of pain: confronting racial and ethnic disparities in pain. Pain Med. 2003;4(3):277-294. doi:10.1046/j.1526-4637.2003.03034.x 12974827

[4] Nelson A. Unequal treatment: confronting racial and ethnic disparities in health care. J Natl Med Assoc. 2002;94(8):666-668. 12152921 PMC2594273

[5] Hatzenbuehler ML, Phelan JC, Link BG. Stigma as a fundamental cause of population health inequalities. Am J Public Health. 2013;103(5):813-821. doi:10.2105/AJPH.2012.301069 23488505 PMC3682466

[6] Link BG, Phelan JC. Conceptualizing stigma. Annu Rev Sociol. 2001;27:363-385. doi:10.1146/annurev.soc.27.1.363

[7] Pescosolido BA, Martin JK. The stigma complex. Annu Rev Sociol. 2015;41:87-116. doi:10.1146/annurev-soc-071312-145702 26855471 PMC4737963

[8] Stepanikova I, Oates GR. Perceived discrimination and privilege in health care: the role of socioeconomic status and race. Am J Prev Med. 2017;52(1S1)(suppl 1):S86-S94. doi:10.1016/j.amepre.2016.09.024 27989297 PMC5172593

[9] Gonzalez D, Skopec L, McDaniel M, Kenney G. Perceptions of discrimination and unfair judgment while seeking health care. Robert Wood Johnson Foundation. Accessed July 31, 2023. https://www.rwjf.org/en/insights/our-research/2021/03/perceptions-of-discrimination-and-unfair-judgment-while-seeking-health-care.html

[10] Beach MC, Saha S, Park J, . Testimonial injustice: linguistic bias in the medical records of Black patients and women. J Gen Intern Med. 2021;36(6):1708-1714. doi:10.1007/s11606-021-06682-z 33754318 PMC8175470

[11] Sun M, Oliwa T, Peek ME, Tung EL. Negative patient descriptors: documenting racial bias in the electronic health record. Health Aff (Millwood). 2022;41(2):203-211. doi:10.1377/hlthaff.2021.01423 35044842 PMC8973827

[12] Himmelstein G, Bates D, Zhou L. Examination of stigmatizing language in the electronic health record. JAMA Netw Open. 2022;5(1):e2144967. doi:10.1001/jamanetworkopen.2021.44967 35084481 PMC8796019

[13] Balderston JR, Gertz ZM, Seedat R, . Differential documentation of race in the first line of the history of present illness. JAMA Intern Med. 2021;181(3):386-388. doi:10.1001/jamainternmed.2020.5792 33427857 PMC7802002

[14] Burgess DJ. Are providers more likely to contribute to healthcare disparities under high levels of cognitive load: how features of the healthcare setting may lead to biases in medical decision making. Med Decis Making. 2010;30(2):246-257. doi:10.1177/0272989X09341751 19726783 PMC3988900

[15] Maina IW, Belton TD, Ginzberg S, Singh A, Johnson TJ. A decade of studying implicit racial/ethnic bias in healthcare providers using the implicit association test. Soc Sci Med. 2018;199:219-229. doi:10.1016/j.socscimed.2017.05.009 28532892

[16] Collins SA, Bakken S, Vawdrey DK, Coiera E, Currie L. Clinician preferences for verbal communication compared to EHR documentation in the ICU. Appl Clin Inform. 2011;2(2):190-201. doi:10.4338/ACI-2011-02-RA-0011 23616870 PMC3631921

[17] Hall WJ, Chapman MV, Lee KM, . Implicit racial/ethnic bias among health care professionals and its influence on health care outcomes: a systematic review. Am J Public Health. 2015;105(12):e60-e76. doi:10.2105/AJPH.2015.302903 26469668 PMC4638275

[18] Sabin JA, Greenwald AG. The influence of implicit bias on treatment recommendations for 4 common pediatric conditions: pain, urinary tract infection, attention deficit hyperactivity disorder, and asthma. Am J Public Health. 2012;102(5):988-995. doi:10.2105/AJPH.2011.300621 22420817 PMC3483921

[19] P Goddu A, O’Conor K, Lanzkron S, . Do words matter: stigmatizing language and the transmission of bias in the medical record. J Gen Intern Med. 2018;33(5):685-691. doi:10.1007/s11606-017-4289-2 29374357 PMC5910343

[20] Johnson AEW, Pollard TJ, Shen L, . MIMIC-III, a freely accessible critical care database. Sci Data. 2016;3:160035. doi:10.1038/sdata.2016.35 27219127 PMC4878278

[21] Gablasova D, Brezina V, Mcenery T, Boyd E. Epistemic stance in spoken L2 English: the effect of task and speaker style. Appl Linguist. 2017;38(5):613-637. doi:10.1093/applin/amv055

[22] Talmina N, Rawlins K. Evidential meaning of English clause-embedding verbs. In: Proceedings of the Annual Meeting of the Cognitive Science Society. 2021. Accessed May 31, 2023. https://escholarship.org/uc/item/64r2h2g3

[23] Papafragou A, Li P, Choi Y, Han CH. Evidentiality in language and cognition. Cognition. 2007;103(2):253-299. doi:10.1016/j.cognition.2006.04.001 16707120 PMC1890020

[24] Yang A. Zheng S-Y, Ge G-C. Epistemic modality in English-medium medical research articles: a systemic functional perspective. English for Specific Purposes. 2015;38:1-10. doi:10.1016/j.esp.2014.10.005

[25] FitzGerald C, Hurst S. Implicit bias in healthcare professionals: a systematic review. BMC Med Ethics. 2017;18(1):19. doi:10.1186/s12910-017-0179-8 28249596 PMC5333436

[26] Zestcott CA, Blair IV, Stone J. Examining the presence, consequences, and reduction of implicit bias in health care: a narrative review. Group Process Intergroup Relat. 2016;19(4):528-542. doi:10.1177/1368430216642029 27547105 PMC4990077

[27] Hertzog WF. A case of puerperal eclampsia. JAMA. 1883;I(7):220-221. doi:10.1001/jama.1883.02390070028009

[28] Ehrlich M. Retention of urine prom supposed double bladder. Boston Med Surg J. 1830;3(22):351-353. doi:10.1056/NEJM183007130032203

[29] Sykes DB, Nichols DN. There is no denying it, our medical language needs an update. J Grad Med Educ. 2015;7(1):137-138. doi:10.4300/JGME-D-14-00332.1 26217448 PMC4507913

[30] Luks AM, Goldberger ZD. Watch your language—misusage and neologisms in clinical communication. JAMA Intern Med. 2021;181(1):5-6. doi:10.1001/jamainternmed.2020.5679 33136114

[31] Windish DM, Price EG, Clever SL, Magaziner JL, Thomas PA. Teaching medical students the important connection between communication and clinical reasoning. J Gen Intern Med. 2005;20(12):1108-1113. doi:10.1111/j.1525-1497.2005.0244.x 16423099 PMC1490291

[32] Haider AH, Sexton J, Sriram N, . Association of unconscious race and social class bias with vignette-based clinical assessments by medical students. JAMA. 2011;306(9):942-951. doi:10.1001/jama.2011.1248 21900134 PMC3684149

[33] Harmon RR, Coney KA. The persuasive effects of source credibility in buy and lease situations. J Mark Res. 1982;19(2):255-260. doi:10.1177/002224378201900209

[34] Hong S, Len-Riós ME. Does race matter: implicit and explicit measures of the effect of the PR spokesman’s race on evaluations of spokesman source credibility and perceptions of a PR crisis’ severity. Journal of Public Relations Research. 2015;27(1):63-80. doi:10.1080/1062726X.2014.929502

[35] Green AR, Carney DR, Pallin DJ, . Implicit bias among physicians and its prediction of thrombolysis decisions for Black and White patients. J Gen Intern Med. 2007;22(9):1231-1238. doi:10.1007/s11606-007-0258-5 17594129 PMC2219763

[36] Sabin JA, Rivara FP, Greenwald AG. Physician implicit attitudes and stereotypes about race and quality of medical care. Med Care. 2008;46(7):678-685. doi:10.1097/MLR.0b013e3181653d58 18580386

[37] van Ryn M, Burke J. The effect of patient race and socio-economic status on physicians’ perceptions of patients. Soc Sci Med. 2000;50(6):813-828. doi:10.1016/S0277-9536(99)00338-X 10695979

[38] Street RL Jr, Gordon H, Haidet P. Physicians’ communication and perceptions of patients: is it how they look, how they talk, or is it just the doctor? Soc Sci Med. 2007;65(3):586-598. doi:10.1016/j.socscimed.2007.03.036 17462801 PMC2811428

[39] Dyrbye L, Herrin J, West CP, . Association of racial bias with burnout among resident physicians. JAMA Netw Open. 2019;2(7):e197457. doi:10.1001/jamanetworkopen.2019.7457 31348503 PMC6661712

[40] Fernández L, Fossa A, Dong Z, . Words matter: what do patients find judgmental or offensive in outpatient notes? J Gen Intern Med. 2021;36(9):2571-2578. doi:10.1007/s11606-020-06432-7 33528782 PMC8390578

[41] Beach MC, Branyon E, Saha S. Diverse patient perspectives on respect in healthcare: a qualitative study. Patient Educ Couns. 2017;100(11):2076-2080. doi:10.1016/j.pec.2017.05.010 28602565 PMC6400635

[42] Weech-Maldonado R, Hall A, Bryant T, Jenkins KA, Elliott MN. The relationship between perceived discrimination and patient experiences with health care. Med Care. 2012;50(9 Suppl 2):S62-S68. doi:10.1097/MLR.0b013e31825fb235 22895233 PMC3726249

[43] Blanchard J, Lurie N. R-E-S-P-E-C-T: patient reports of disrespect in the health care setting and its impact on care. J Fam Pract. 2004;53(9):721-730. 15353162

[44] Klinger EV, Carlini SV, Gonzalez I, . Accuracy of race, ethnicity, and language preference in an electronic health record. J Gen Intern Med. 2015;30(6):719-723. doi:10.1007/s11606-014-3102-8 25527336 PMC4441665

