# Supplementary material for: Race and Ethnicity and Clinician Linguistic Expressions of Doubt in Hospital Admission Notes
Source: JAMA Netw Open. 2024 Oct 14;7(10):e2438550. doi: 10.1001/jamanetworkopen.2024.38550 (PMC11581534; doi:10.1001/jamanetworkopen.2024.38550)
Supplement: Supplement 1. — eFigure 1. Annotation Guide for Clinician Doubt in Admission Notes eTable 1. Extraction Details and Examples for Evidential and Epistemic Modality Terms eTable 2. Accuracy Rate and Percent Agreement for a Random Set of Text Samples for Each Term eFigure 2. Approach to Semantic Annotation of Epistemic Stance Terms in Health Records eFigure 3. Overall Percentage Agreement and Intercoder Reliability of Random Text Samples eTable 3. List of Stop Words Used in Regression Estimates of Falsification Test eFigure 4. Flow Diagram from Sample Population to Study Population eTable 4. Regression Estimates of Unadjusted and Adjusted Odds Ratios Between Patient Characteristics and the Presence of Any Doubt Language in Admission Notes, by Hospital Site eTable 5. Unadjusted Odds Ratios of Occurrence of Evidential and Epistemic Modality by Race eTable 6. Subgroup Analysis of Intensive Care Unit Documentation: Patient, Encounter, and Admission Note Characteristics by Hospital Site eTable 7. Subgroup Analysis of Intensive Care Unit (ICU) Documentation: Number of Times Doubt Language Occurred in ICU Admission Notes by Hospital Site eTable 8. Subgroup Analysis of Intensive Care Unit Documentation: Regression Estimates of Unadjusted and Adjusted Odds Ratios Between Patient Characteristics and the Presence of Any Doubt Language in Admission Notes by Hospital Site eFigure 5. Subgroup Analysis of Intensive Care Unit Documentation: Unadjusted and Adjusted Associations Patient Race and Ethnicity and the Presence of Doubt Language by Hospital Site eTable 9. Unadjusted and Adjusted Regression Estimates of Patient Characteristics and Occurrences of Doubt Language eTable 10. Falsification Test: Unadjusted and Adjusted Regression Estimates of Patient Characteristics and Rate Ratios of Stop Words [file jamanetwopen-e2438550-s001.pdf]

Supplemental Online Content

Lee CR, Aysola J, Chen X, et al. Race and ethnicity and clinician linguistic expressions of doubt in hospital admission notes. *JAMA Netw Open*. 2024;7(10):e2438550. doi:10.1001/jamanetworkopen.2024.38550

eFigure 1. Annotation Guide for Clinician Doubt in Admission Notes

eTable 1. Extraction Details and Examples for Evidential and Epistemic Modality Terms

eTable 2. Accuracy Rate and Percent Agreement for a Random Set of Text Samples for Each Term

eFigure 2. Approach to Semantic Annotation of Epistemic Stance Terms in Health Records

eFigure 3. Overall Percentage Agreement and Intercoder Reliability of Random Text Samples

eTable 3. List of Stop Words Used in Regression Estimates of Falsification Test

eFigure 4. Flow Diagram from Sample Population to Study Population

eTable 4. Regression Estimates of Unadjusted and Adjusted Odds Ratios Between Patient Characteristics and the Presence of Any Doubt Language in Admission Notes, by Hospital Site

eTable 5. Unadjusted Odds Ratios of Occurrence of Evidential and Epistemic Modality by Race

eTable 6. Subgroup Analysis of Intensive Care Unit Documentation: Patient, Encounter, and Admission Note Characteristics by Hospital Site

eTable 7. Subgroup Analysis of Intensive Care Unit (ICU) Documentation: Number of Times Doubt Language Occurred in ICU Admission Notes by Hospital Site

eTable 8. Subgroup Analysis of Intensive Care Unit Documentation: Regression Estimates of Unadjusted and Adjusted Odds Ratios Between Patient Characteristics and the Presence of Any Doubt Language in Admission Notes by Hospital Site

eFigure 5. Subgroup Analysis of Intensive Care Unit Documentation: Unadjusted and Adjusted Associations Patient Race and Ethnicity and the Presence of Doubt Language by Hospital Site

eTable 9. Unadjusted and Adjusted Regression Estimates of Patient Characteristics and Occurrences of Doubt Language

eTable 10. Falsification Test: Unadjusted and Adjusted Regression Estimates of Patient Characteristics and Rate Ratios of Stop Words

This supplemental material has been provided by the authors to give readers additional information about their work.

© 2024 Lee CR et al. *JAMA Network Open.*

eFigure 1. Annotation Guide for Clinician Doubt in Admission Notes

ANNOTATION GENERAL RULES

1. Annotate phrases that pertain to patterns in language suggesting doubt in patient reports.
   Definition:
   a. Epistemic stance: attitudes, feelings, judgments, or commitment concerning the propositional content of a message
      a. Source (i.e., holder of the attitude) - clinician perspective
      b. Aspect (i.e., target of the attitude) - patient history includes symptoms, behaviors, and adherence or response to treatment
      c. Type of attitude - Language indicating the origin of information relative to the source (i.e., clinician perspective) or language indicating degree of confidence in the perceived validity of patients' symptoms or treatment adherence

   Some general examples:
   a. Patient denies shortness of breath.
   b. Patient complains of chest pain lasting four hours.
   c. Patient claims he stopped drinking 6 weeks ago.

Mark YES=1, if the word or phrase meets criteria for epistemic stance, NO=0 if the word or phrase DOES NOT meet criteria

2. Mark 'NO=0' for words or phrases that refer to physical or emotional symptoms of people other than the patient, **even if the caregiver (e.g. nurse, other staff) or family member provided history on behalf of the patient's symptoms or behaviors**
   a. Example of what NOT to include: She spoke with the patient today and denies he was short of breath.

3. If it is uncertain whether the word or phrase is in reference to the patient's reported symptoms or behaviors, **then Mark 'YES=1' if it is reasonable to infer that the information was obtained from the patient based on the context of the snippet.**

4. Mark 'NO=0' for words or phrases that refer to a medical condition, prior history from other notes, pathophysiologic processes mental/physical states or **acceptance/refusal of treatment and/or procedures.**
   a. Examples of what NOT to include:
      i. Patient seems euvolemic.
      ii. Patient insists on taking medication as previously describes.
      iii. Others: Immunocompromised state, usual state of health, based on prior notes

5. Mark 'NO=0' for words or phrases that refer to patient preferences for treatment or advanced care directives.

6. For duplicated text:
   a. If you annotate a phrase and see it duplicated later for the same word, you should rate it (the same) again.
   b. HOWEVER, if you see the snippet later in reference to a different word, then rate it again, but in accordance to how it fits for the new word being annotated.

© 2024 Lee CR et al. *JAMA Network Open.*

eTable 1. Extraction Details and Examples for Evidential and Epistemic Modality Terms

Terms	Extraction Details	Revised Extraction Details	Example –To Included	Example—To NOT Included
Complains	Included all counts of “complains” + “complained” + “complaining”	Above 80% accuracy rate - no change	He was complaining of some abdominal pain<br><br>He also complains of shortness of breath	The patient has multiple complaints
Denies	Included all counts of “denies” + “denied” + “denying”	Above 80% accuracy rate - no change	He denies that this as a barrier to adherence<br><br>He denied current suicidal thoughts<br><br>She also denies any major changes to her diet	..referred to skilled nursing, but denied by insurance
Endorses	Included all counts of “endorses” + “endorsed” + “endorsing”	Above 80% accuracy rate - no change	He endorses weakness/light-headedness<br><br>She endorsed shortness of breath<br><br>Patient also endorses decreased appetite	--
Notes	Included all counts of “notes” and “noting”<br><br>Do NOT include “noted” as majority of instances are likely phrases where the patient was not the person who did the noting, such as “From EMS, he was noted to have…”	Below 80% accurate rate - Revised extraction:<br><br>Include all counts of “patient/pt notes” + “she/he/who/also notes”	He notes the dyspnea remains present<br><br>Pt also noted that her nausea has increased	Per chart/prior notes<br><br>Technician notes from<br><br>ED notes pt to have rales at the bases.

© 2024 Lee CR et al. *JAMA Network Open.*

Reports	Included all counts of “reports” and “reporting”<br><br>Do NOT include “reported” as majority of instances may be embedded within template text (e.g. when lab or medication was “Reported on DATE”	Above 80% accuracy rate - no change	She reports she is feeling much better<br><br>The patient reports taking his lasix regularly<br><br>She reports that she had an EGD a few years ago	Based on prior reports
Says	Included all counts of “says” and “saying”<br><br>Do NOT include “said” as majority of instances may refer to NSAIDS	Below 80% accurate rate - Revised extraction:<br><br>Include all counts of “says” + “saying that/she/he”	She says that she is hungry<br><br>He says the rash spread from his arm to his torso<br><br>She says that Tylenol does not help her pain	I counseled him saying that..<br><br>Prior notes say that he took…
States	Include all counts of “state” + “states” + “stated”	Below 80% accurate rate - Revised extraction:<br><br>Include all counts of “states” AND Exclude all counts of “state” + “stated”	Pt states that he has been asymptomatic<br><br>He stated that his last drink was yesterday<br><br>Guardian stated the patient felt dizzy	Usual state of health<br><br>Immunocompromised state<br><br>Hypo(er)volemic state
Thinks	Include all counts of “thinks” and “thought”	Below 80% accurate rate - Revised extraction:<br><br>Include all counts of “Patient/pt thinks” + “Patient/pt thought”	She thinks that she has a UTI<br><br>He thought he took his anti-seizure medicine yesterday	thought to be symptomatic bradycardia (or other pathophysiologic process)

© 2024 Lee CR et al. *JAMA Network Open.*

Describes	Include all counts of “describe” + “describes” + “described”	Below 80% accurate rate - Revised extraction:<br><br>Include all counts of “describes” + “describe”<br>Exclude all counts of “described”	Patient describes nausea and vomiting since Friday<br><br>She described her chest pain as constant overnight	Right upper lobe nodular density as described in the prior CT<br><br>Explain the finding described in the echocardiogram
Express	Include all counts of “express” + “expresses” + “expressed”	Below 80% accurate rate – no revisions to extraction as majority of occurrences refer to goals of care	He expresses that he has been having difficulty with gate<br><br>Patient has wanted DNI but expressed SI, to discuss with psych	It has been expressed that he must stop smoking<br><br>expressed wishes are for DNR/DNI status.<br><br>but clearly against patient's previously expressed wishes
Mentions	Include all counts of “mentions” + “mentioned” + “mention”	Below 80% accurate rate – Revised extraction:<br><br>Include all counts of “patient/pt/she/he mentions” + “patient/pt/she/he mentioned” + “who mentioned” +<br>“also mentioned” + “does/did mention”	she developed nausea and vomiting, which she mentions ""felt like DKA""<br><br>he does mention having this productive cough at baseline	As mentioned, patient has a history of GIB<br><br>Records mention ARDS, but not intubated.
Argues	Include all counts of “argue” + “argues” + “argued”	Below 80% accurate rate – Revised extraction:<br><br>Include all counts of “patient/pt/he/she argued”	She argued that her last treatment led to a rash<br><br>Pt argued that he took his meds prior to ED	Pt may have sarcoid flair, although acute onset argues against this<br><br>Would argue a tagged RBC scan might at least tell us where
Tells me	Includes all counts of “tells” + “told” + “telling” (+me)	Below 80% accurate rate – Revised extraction:<br><br>Include all counts of “tells me” + “told me”<br>Exclude all counts of “told me to”as not related to patient report	pt also tells me he has h/o withdrawal.<br><br>Patient tells me that is chest pain is burning in quality	he tells me that she "has made it clear she wants nothing to …”

© 2024 Lee CR et al. *JAMA Network Open.*

Explains	Include all counts of "explain" + "explains" + "explained"	Below 80% accurate rate – Revised extraction:<br><br>Include all counts of "patient/pt/she/he explains/explained"<br><br>Exclude all counts of "explain" as majority relate to clinical assessment	She explains that she felt dizzy prior…	Cardiology explained that…<br><br>Pulmonary embolism explains new onset...
Assumes	Include all counts of "assume" + "assumes" + "assumed"	Below 80% accurate rate – Revised extraction:<br><br>Include all counts of "patient/pt/she/he assumes/assumed"<br><br>Exclude all counts of "assume" as majority relate to clinical assessment	She assumed her symptoms at that time were due to the flu	Cardiac index was normal based on an assumed oxygen consumption index<br><br>it is reasonable to assume that he has endovascular / endocarditis<br><br>Reasonable to assume the EF will get better
Claims	Include all counts of "claim" + "claims" + "claimed"	Below 80% accurate rate – Revised extraction:<br><br>Include all counts of "claims" + "claimed"	He claims to drink 1 glass a night<br><br>She claimed that she has been depressed for 5 years<br><br>Wife claims he was making odd noises	She is waiting to her insurance to process her claim(s)
Expects	Include all counts of "expect" + "expects" + expected	Below 80% accurate rate – Revised extraction:<br><br>Include all counts of "patient/pt/he/she expected/expects"	Husband expected pt's blurry vision to improve<br><br>She expected to have less pain on medication	Expected to make full recovery<br><br>Expect this due to nonadherence<br><br>Expected life expectancy

© 2024 Lee CR et al. *JAMA Network Open.*

		Exclude all counts of “expect” as majority related to clinical assessment		Would [not] expect [dx, physical exam, tx response]…
Implies	Include all counts of “implies” + “imply” + “implied”	Below 80% accurate rate – Revised extraction:<br><br>Include all counts of “patient/pt/he/she implies/implied”	He implied that her last drug use was last night	Given preserved oxygenation this implies that there is no longer bloodflow to this lung<br><br>he has lower extremity edema, which may imply some acute on chronic heart failure
Suggests	Include all counts of “suggest” + “suggests” + “suggested”	Below 80% accurate rate – Revised extraction:<br><br>Include all counts of “Patient/pt/he/she suggests/suggested”<br><br>Exclude all counts of “suggest” as majority related to clinical assessment	She suggested that she drinks daily<br><br>He suggested that this pain may have preceded the fall..	Suggests pt had problem for years<br><br>Suggests that pt has IBD<br><br>CXR suggests infection/pneumonia
Suspects	Include all counts of “suspect” + “suspects” + “suspected”	Below 80% accurate rate – Revised extraction:<br>Include all counts of “Patient/pt/he/she suspects/suspected”<br><br>Exclude all counts of “suggest” as majority related to clinical assessment	He suspects the Chinese food led to…<br><br>She suspected that rash would resolve<br><br>He suspected that his mother was having a stroke	Suspect this is related to CHF<br><br>Cardiology suspects patient will respond to BB
Insists	Include all counts of “insist” + “insists” + “insisted”	Below 80% accurate rate – no changes as majority of occurrences refer to acceptance or refusal of treatment/procedure	She insists that the pain is not improving<br><br>He insisted that he hadn’t coughed	I/We insisted…<br><br>Surgery insists on having pre-op eval

© 2024 Lee CR et al. *JAMA Network Open.*

Believes	Include all counts of “believe” + “believed” + “believes”	Below 80% accurate rate – Revised extraction:<br><br>Include all counts of “Patient/pt/she/he believes”<br><br>Exclude all counts of “believe” + “believed” as majority related to clinical assessment	No prodrome but he believes that he lost consciousness<br><br>He believes the morphine relieved the chest pain	With CVP of 20, believed to be due to redistribution of fluids<br><br>altered mental status believed to be due to a combination of UTI, narcotics,
Feels	Include all counts of “feel” + “felt” + “feels”	Below 80% accurate rate – no changes as majority of occurrences refer to physical state or physical assessment	She notes that she feels as if she has a weak cough<br><br>He feels that he is improved	lesion felt to be too large to be amenable to stenting<br><br>he became sedated which was felt to have led to an aspiration event
Adamant	Include all counts of “adamant”	Below 80% accurate rate – Revised extraction:<br><br>Include all counts of “pt/patient/she/he is adamant”<br><br>Exclude all counts of “adamantly”	He adamantly denies recent alcohol use<br><br>Adamant that his leg pain is 10/10	Ortho [other MD] adamant this is not…
Possibly	Include all counts of “possibly” + “possible””	Below 80% accurate rate – no changes as majority of occurrences related to clinical assessment	Denies any abdominal pain, f/c, possibly a small amount of diarrhea.	she was witnessed to have generalized seizure possibly several times.<br><br>CXR showing possible new infiltrate concerning for a pneumonia
Probably	Include all counts of “probably” + “probable”	Below 80% accurate rate – no changes as majority of occurrences related to clinical assessment	She states that she will probably drink again..<br><br>He states his pain probably started last night	Probably explaining why…<br><br>Probably due to infection

© 2024 Lee CR et al. *JAMA Network Open.*

Perhaps	Include all counts of "perhaps"	Below 80% accurate rate – no changes as majority of occurrences related to clinical assessment	he has not had a drink for at least 8 days and perhaps longer	his would leave aspiration as perhaps most likely diagnosis<br><br>increased drive to breathe, perhaps related to stimulation of pulmonary receptors.
Maybe	Include all counts of "maybe"	Below 80% accurate rate – no changes as majority of occurrences related to clinical assessment	increased frequency of BMs over the weekend, maybe up to [**2-25**] (baseline [**1-27**] daily).	likely dependent on atrial kick which maybe worsening heart failure<br><br>Severe hypoventilation likely due to COPD and maybe component of obesity<br><br>Cardiac MRI maybe better at delineate the ventricular septal
According to	Include all counts of "according to"	Below 80% accurate rate – Revised extraction:<br><br>Include all counts of "according to patient"	History of right breast calcification, stable according to patient by mammogram over the past 3 years<br><br>Patient describes associated fevers (according to patient 102)	However, according to his home nurse, his exercise tolerance has..<br><br>according to EMS report, had just been committed for rehab
Apparently	Include all counts of "apparently" + "apparent"	Below 80% accurate rate – Revised extraction:<br><br>Include all counts of "with/who apparently"<br><br>Exclude all counts of "apparent"	He apparently vomited 10x at home<br><br>Apparently she has not used cocaine in 5 years<br><br>He apparently endorsed suicidality at another hospital…	Apparently there is involvement of R ovary as well<br><br>Apparent swelling on exam<br><br>Adamantly refused…[treatment or advanced directive]

© 2024 Lee CR et al. *JAMA Network Open.*

Supposedly	Include all counts of "supposedly"	Below 80% accurate rate – Revised extraction:<br><br>Include all counts of "she/he/patient/pt supposedly"	She supposedly had a bad dream, fell, and hit her head on the<br><br>Baseline tachycardia--supposedly has baseline HR in 100's for year	Oxygen sat was supposedly 60%<br><br>This hematoma was supposedly removed by vascular surgery
Reportedly	Include all counts of "reportedly"	Below 80% accurate rate – Revised extraction:<br><br>Include all counts of "Patient/pt/she/he reportedly"	Patient was reportedly asymptomatic.<br><br>reportedly felt well this morning though c/o intermittent di<br><br>She reportedly had abrupt onset of nausea, belching, sweating	CT head reportedly normal.<br><br>Blood pressure reportedly dipped to systolics in 70's<br><br>Cardiomegaly was noted by CXR but bedside echo reportedly showed trace pericardial effusion.
Per patient	Include all counts of "per patient" + "per pt"	Above 80% accurate rate - no change	no rigidity or rebound, per patient at baseline chronic pain.<br><br>and usual heart rates are in the 90s-100s per patient.	last catheterization about 12 years ago per patient's daughter<br><br>Unclear etiology per patient's wife

© 2024 Lee CR et al. *JAMA Network Open.*

eTable 2. Accuracy Rate and Percent Agreement for a Random Set of Text Samples for Each Term

Terms	N	Accuracy Rate[a]	% Agreement[b]	REVISED[c] N	REVISED Accuracy Rate[a]	REVISED % Agreement[b]	Included in Final Analysis
Complains	25	100%	97.33%	--	--	--	Yes
Denies	25	100%	100%	--	--	--	Yes
Endorses	25	100%	100%	--	--	--	Yes
Notes	25	40%	100%	25	92%	88%	Yes
Reports	25	92%	97.33%	--	--	--	Yes
Says	25	76%	84%	25	88%	92%	Yes
States	25	60%	100%	25	88%	96%	Yes
Thinks	25	4%	89.33%	9	100%	100%	No
Describes	25	52%	81.33%	25	96%	76%	Yes
Express	25	8%	78.67%	0	--	--	No
Mentions	25	20%	86.67%	18	77%	83%	Yes
Argues	25	0%	97.33%	0	--	--	No
Tells me	12	66.66%	94.44%	11	76.92%	92.31%	No
Explains	25	0%	100%	1	100%	100%	No
Assumes	25	4%	100%	0	--	--	No
Claims	25	72%	94.67%	25	92%	100%	Yes
Expects	25	0%	100%	1	100%	100%	No
Implies	19	0%	89.47%	0	--	--	No
Suggests	25	0%	97.33%	1	0	100%	No
Suspects	25	0%	100%	6	16.67%	100%	No
Insists	17	23.52941%	84.31%	--	--	--	No
Believes	25	24%	92.00%	25	92%	88%	Yes
Feels	25	10%	76.00%	--	--	--	No
Probably	25	0%	100%	--	--	--	No
Adamant	14	7.142857%	82.46%	1	100%	100%	No
Possibly	25	4%	97.33%	--	--	--	No
Perhaps	25	4%	84.00%	--	--	--	No

© 2024 Lee CR et al. *JAMA Network Open.*

Maybe	25	4%	84.00%	--	--	--	No
According to	25	20%	94.67%	25	68%	96%	Yes
Apparently	25	20%	81.33%	8	37.5%	75%	No
Supposedly	12	33.33%	83.33%	1	100%	100%	No
Allegedly	0	--	--	--	--	--	No
Reportedly	25	32%	70.67%	0	--	--	No
Per patient	25	80%	89.33%	--	--	--	Yes

[a]Accuracy rate: Number of true positives for each term (as determined by annotator consensus) divided by number of occurrences for each term in random sample (True Positives[TP]/N)

[b]Percent agreement: Number of true positive and true negative for each term (as determined by annotator consensus) divided by number of occurrences for each term in random sample (True positive [TP] + True Negative [TN])/N)

[c]Revised: For terms that did not meet the threshold of at least 80% accuracy rate in random text samples for each term, the regular expressions were revised and a repeated set of random text samples were abstracted and annotated.

© 2024 Lee CR et al. *JAMA Network Open.*

eFigure 2. Approach to Semantic Annotation of Epistemic Stance Terms in Health Records

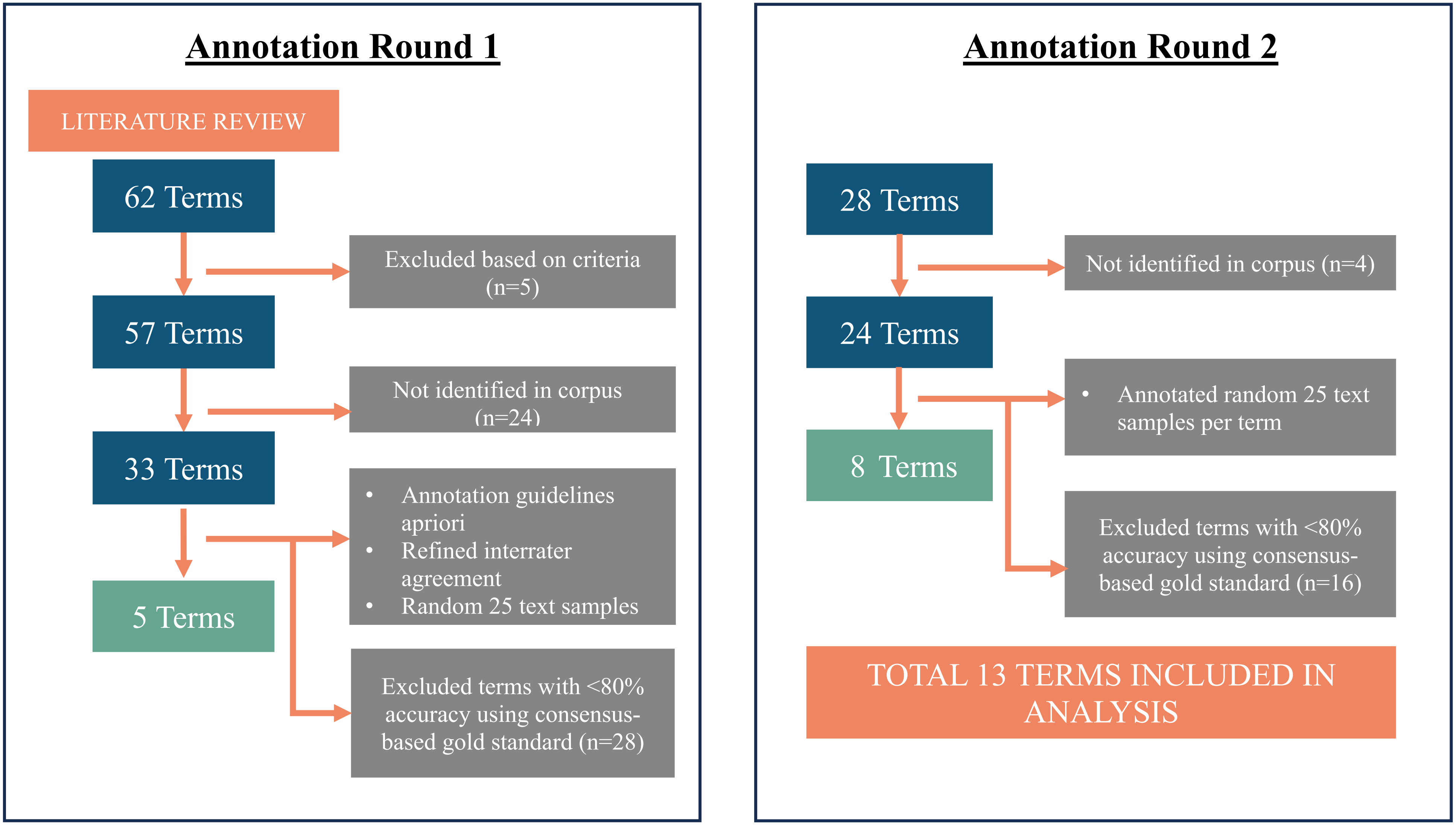

To identify the words or phrases reflecting epistemic stance in admission notes, we manually assembled a list of terms based on expert opinion and published sources, drawing from existing medical literature on stigmatizing language and computational linguistics literature. We identified a total of 62 epistemic stance words or phrases that were relevant to the text of health records.

We used regular expressions, an exact string-matching technique, to capture all the relevant words or phrases within the Medical Information Mart for Intensive Care III (MIMIC-III) corpus. To identify words in all relevant tenses, we accounted for variation in verb tense by including the simple present, simple past, and present participle tenses of a verb form (e.g. denies, denied, denying). We detected 33 of these words or phrases within the MIMIC-III corpus. To ensure each word or phrase accurately reflected epistemic stance (Table 1) when used in context, we abstracted a random set of 25 text samples for each word and 3 team members (C.R.L., E.A., A.K.) independently coded whether the word or phrase represented epistemic stance using annotation guidelines established a priori (eFigure 2). For instances where coders did not agree, a consensus decision about whether the text sample reflected epistemic stance was determined by group review.

To create a final, validated list of words and phrases, we included those that were identified as epistemic stance with ≥80% of accuracy rate (i.e., of the random text samples identified for a given term, raters agreed that the term represented epistemic stance in 80% of the text samples). For certain terms that did not reach this threshold, we conducted a second round of annotation in which we revised the regular expressions to include preceding pronouns or exclude variation of the words that related to medical reasoning or treatment preferences (eTable 1). We repeated an abstraction of a random set of 25 text samples, which were independently coded by each team member. Terms were then eliminated if they did not meet the threshold ≥80% accuracy rate or had less than 10 string matches for the random set of text samples (eTable 2). Thirteen terms were included in the final analysis (Table 1). For all terms, percent agreement (i.e., proportion of agreement on coded words or phrases between independent coders) was between 86.9-92.5% and intercoder reliability was 0.78 (eFigure 3).

© 2024 Lee CR et al. *JAMA Network Open.*

eFigure 3. Overall Percent Agreement and Intercoder Reliability of Random Text Samples

	CRL & EA	CRL & AK	AK & EA
**% Agreement**	92.54%	89.93%	86.94%
**Kappa Score**	0.8403	0.779	0.7113

	Coef.	Std. Err.	95% Conf. Interval	
**Cohen/Conger's Kappa**	0.7775	0.0182	0.7418	0.8133
**Scott/Fleiss Kappa**	0.7773	0.0183	0.7414	0.8131

© 2024 Lee CR et al. *JAMA Network Open.*

eTable 3. List of Stop Words[a] Used in Regression Estimates of Falsification Test

Stop words	'i', 'me', 'my', 'myself', 'we', 'our', 'ours', 'ourselves', 'you', "you're", "you've", "you'll", "you'd", 'your', 'yours', 'yourself', 'yourselves', 'he', 'him', 'his', 'himself', 'she', "she's", 'her', 'hers', 'herself', 'it', "it's", 'its', 'itself', 'they', 'them', 'their', 'theirs', 'themselves', 'what', 'which', 'who', 'whom', 'this', 'that', "that'll", 'these', 'those', 'am', 'is', 'are', 'was', 'were', 'be', 'been', 'being', 'have', 'has', 'had', 'having', 'do', 'does', 'did', 'doing', 'a', 'an', 'the', 'and', 'but', 'if', 'or', 'because', 'as', 'until', 'while', 'of', 'at', 'by', 'for', 'with', 'about', 'against', 'between', 'into', 'through', 'during', 'before', 'after', 'above', 'below', 'to', 'from', 'up', 'down', 'in', 'out', 'on', 'off', 'over', 'under', 'again', 'further', 'then', 'once', 'here', 'there', 'when', 'where', 'why', 'how', 'all', 'any', 'both', 'each', 'few', 'more', 'most', 'other', 'some', 'such', 'no', 'nor', 'not', 'only', 'own', 'same', 'so', 'than', 'too', 'very', 's', 't', 'can', 'will', 'just', 'don', "don't", 'should', "should've", 'now', 'd', 'll', 'm', 'o', 're', 've', 'y', 'ain', 'aren', "aren't", 'couldn', "couldn't", 'didn', "didn't", 'doesn', "doesn't", 'hadn', "hadn't", 'hasn', "hasn't", 'haven', "haven't", 'isn', "isn't", 'ma', 'mightn', "mightn't", 'mustn', "mustn't", 'needn', "needn't", 'shan', "shan't", 'shouldn', "shouldn't", 'wasn', "wasn't", 'weren', "weren't", 'won', "won't", 'wouldn', "wouldn't"

[a]Stop words refers to a set of commonly used words in language that do not add additional meaning to a sentence, and therefore, are traditionally ignored or removed during processing of test. We used the Natural Language Toolkit in Python to assemble this list of stop words.

© 2024 Lee CR et al. *JAMA Network Open.*

eFigure 4. Flow Diagram from Sample Population to Study Population

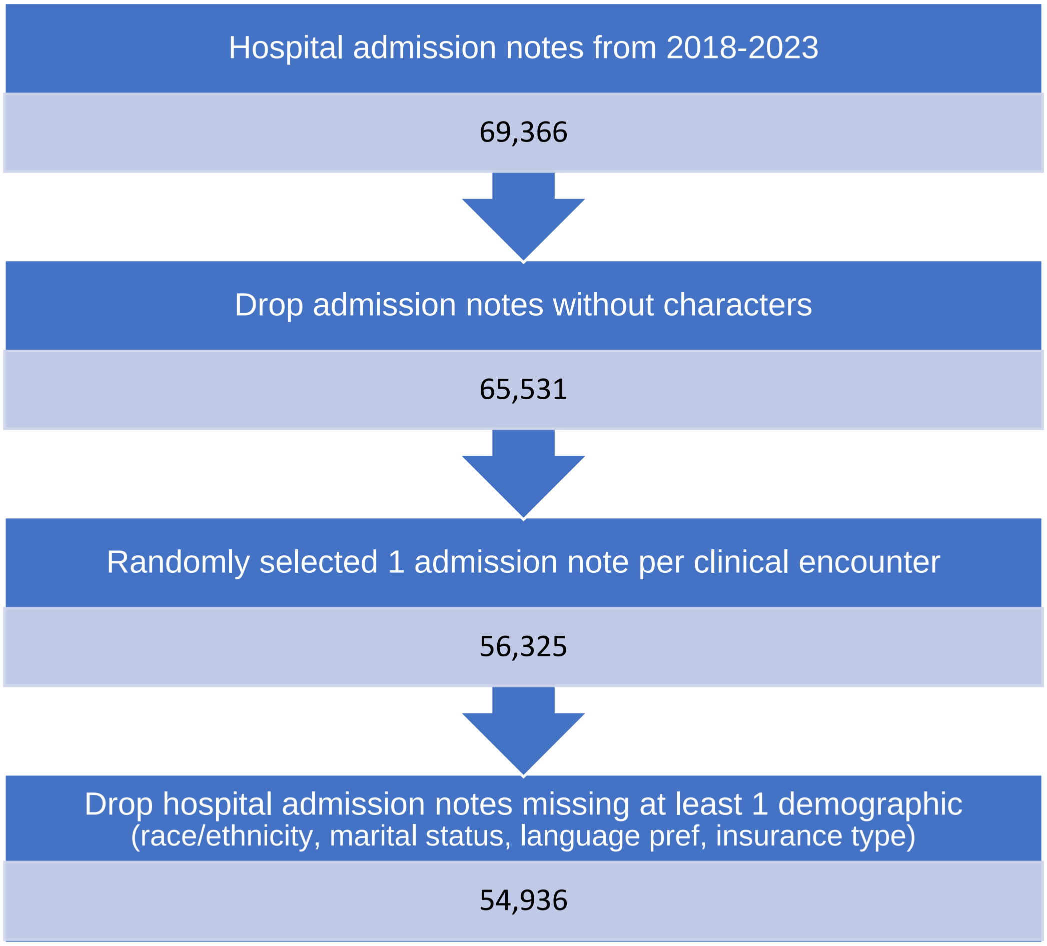

	Hospitals A-C Combined	Hospital A	Hospital B	Hospital C	MIMIC III
**Total number of admission notes**	54936	24783	15744	14409	5649
**Number of unique patients**	N/A	N/A	N/A	N/A	4675
**Number of unique clinicians**	3605	2304	1001	1788	396

© 2024 Lee CR et al. *JAMA Network Open.*

eTable 4. Regression Estimates of Unadjusted and Adjusted Odds Ratios between Patient Characteristics and the Presence of Any Doubt Language[a] in Admission Notes, by Hospital Site

	HOSPITALS A-C Combined			HOSPITAL A			HOSPITAL B			HOSPITAL C		
**Characteristics**	**Unadjusted OR[b] (95%CI)**	**Adjusted OR[b,c] (95%CI)**	**Adjusted OR[b,c,d] (95%CI)**	**Unadjusted OR[b] (95%CI)**	**Adjusted OR[b,c] (95%CI)**	**Adjusted OR[b,c,d] (95%CI)**	**Unadjusted OR[b] (95%CI)**	**Adjusted OR[b,c] (95%CI)**	**Adjusted OR[b,c,d] (95%CI)**	**Unadjusted OR[b] (95%CI)**	**Adjusted OR[b,c] (95%CI)**	**Adjusted OR[b,c,d] (95%CI)**
**Age**	1 [1,1]	1 [1,1]	1 [1,1]	1 [0.99,1]	1 [1,1]	1 [1,1]	1 [1,1]	1 [1,1.01]	1 [1,1.01]	1 [0.99,1]	1 [0.99,1]	1 [0.99,1]
	0.03	0.44	0.47	<0.001	0.4	0.41	0.55	0.07	0.13	0.004	0.32	0.34
**Female**	0.99 [0.94,1.05]	0.95 [0.9,1]	0.92 [0.85,0.98]	1.07 [0.99,1.17]	1.01 [0.93,1.1]	0.98 [0.88,1.08]	0.99 [0.87,1.14]	0.96 [0.84,1.11]	0.9 [0.76,1.07]	1.01 [0.92,1.11]	0.98 [0.89,1.08]	0.99 [0.85,1.16]
	0.74	0.07	0.02	0.09	0.78	0.65	0.92	0.6	0.24	0.82	0.7	0.92
**Non-Hispanic Black (NHB)**	1.37 [1.3,1.44]	1.21 [1.14,1.28]	1.15 [1.06,1.25]	1.3 [1.2,1.4]	1.16 [1.06,1.26]	1.11 [0.98,1.26]	1.72 [1.55,1.9]	1.32 [1.17,1.49]	1.14 [0.93,1.4]	1.36 [1.22, 1.51]	1.32 [1.17,1.48]	1.31 [1.14, 1.5]
	<0.001	<0.001	0.001	<0.001	0.001	0.1	<0.001	<0.001	0.2	<0.001	<0.001	<0.001
**POC (excluding NHB)**	0.95 [0.86,1.05]	0.98 [0.88,1.09]	1 [0.84,1.19]	1.02 [0.88,1.18]	1.07 [0.92,1.25]	0.94 [0.74,1.19]	0.88 [0.74,1.06]	0.8 [0.66,0.98]	0.95 [0.62,1.46]	0.91 [0.71,1.16]	0.97 [0.75,1.27]	1.18 [0.83,1.67]
	0.3	0.74	0.97	0.8	0.38	0.59	0.17	0.03	0.81	0.45	0.83	0.37
**Female * Non-Hispanic Black**			1.09 [0.99,1.21]			1.07 [0.92,1.26]			1.23 [0.97,1.55]			1.01 [0.83,1.23]
			0.08			0.37			0.09			0.92
**Female * POC (excluding NHB)**			0.99 [0.8,1.22]			1.25 [0.92,1.68]			0.83 [0.52,1.31]			0.66 [0.4,1.08]
			0.91			0.15			0.42			0.1
**Not English**	0.69 [0.59,0.82]	0.74 [0.62,0.89]	0.74 [0.62,0.89]	0.61 [0.48,0.77]	0.62 [0.48,0.8]	0.63 [0.49,0.81]	0.88 [0.62,1.24]	1.07 [0.74,1.57]	1.05 [0.72,1.54]	0.61 [0.43,0.88]	0.71 [0.49,1.05]	0.73 [0.5,1.08]
	<0.001	0.001	0.001	<0.001	<0.001	<0.001	0.45	0.71	0.79	0.008	0.09	0.12
**Married/Life Partner**	0.77 [0.74,0.81]	0.89 [0.85,0.94]	0.9 [0.85,0.94]	0.79 [0.74,0.84]	0.89 [0.83,0.97]	0.89 [0.83,0.97]	0.59 [0.53,0.65]	0.78 [0.7,0.87]	0.78 [0.7,0.88]	0.9 [0.82,1]	1.02 [0.92,1.14]	1.02 [0.92,1.14]
	<0.001	<0.001	<0.001	<0.001	0.005	0.005	<0.001	<0.001	<0.001	0.04	0.68	0.7
**Medicaid (ref = Medicare)**	1.25 [1.17,1.33]	1.11 [1.02,1.21]	1.11 [1.02,1.2]	1.26 [1.14,1.39]	1.07 [0.94,1.21]	1.06 [0.94,1.21]	1.31 [1.12,1.53]	1.13 [0.94,1.35]	1.11 [0.92,1.33]	1.27 [1.11,1.44]	1.09 [0.93,1.28]	1.09 [0.93,1.28]
	<0.001	0.01	0.02	<0.001	0.31	0.34	0.001	0.21	0.28	<0.001	0.28	0.28
**Employerbased /private/self-pay (ref = Medicare)**	0.8 [0.76,0.85]	0.83 [0.77,0.89]	0.83 [0.77,0.89]	0.91 [0.84,0.99]	0.88 [0.8,0.98]	0.89 [0.8,0.98]	0.58 [0.51,0.67]	0.66 [0.56,0.77]	0.66 [0.56,0.78]	0.88 [0.79,1]	0.85 [0.74,0.97]	0.85 [0.74,0.97]

© 2024 Lee CR et al. *JAMA Network Open.*

	<0.001	<0.001	<0.001	0.04	0.02	0.02	<0.001	<0.001	<0.001	0.04	0.01	0.01
**Self-Discharge (ie., left against medical advice**	1.6 [1.27,2.01]	1.41 [1.12,1.79]	1.42 [1.13,1.8]	1.6 [1.11,2.32]	1.41 [0.97,2.04]	1.41 [0.97,2.05]	1.91 [1.1,3.31]	1.68 [0.97,2.92]	1.69 [0.97,2.93]	1.53 [1.05,2.22]	1.4 [0.96,2.04]	1.4 [0.96,2.04]
	<0.001	0.004	0.003	0.01	0.07	0.07	0.02	0.07	0.06	0.03	0.08	0.09
**Timing of encounter On or after March 1,2020**	0.91 [0.86,0.97]	0.9 [0.84,0.95]	0.89 [0.84,0.95]	0.77 [0.7,0.84]	0.77 [0.7,0.84]	0.77 [0.7,0.84]	1.3 [1.14,1.47]	1.25 [1.1,1.42]	1.25 [1.1,1.42]	0.92 [0.81,1.03]	0.91 [0.8,1.02]	0.91 [0.8,1.02]
	0.003	<0.001	<0.001	<0.001	<0.001	<0.001	<0.001	0.001	0.001	0.15	0.11	0.11
**HOSPITAL B (ref = A)**	1.36 [1.21,1.53]	1.35 [1.2,1.53]	1.36 [1.2,1.53]									
	<0.001	<0.001	<0.001									
**HOSPITAL C (ref = A)**	1.21 [1.11,1.31]	1.11 [1.02,1.21]	1.12 [1.03,1.22]									
	<0.001	0.02	0.01									
**Elixhauser comorbidity index**	1 [0.99,1]	1 [0.99,1]	1 [0.99,1]	0.99 [0.99,1]	1 [0.99,1]	1 [0.99,1]	0.99 [0.98,1]	0.99 [0.98,1]	0.99 [0.98,1]	0.99 [0.99,1]	0.99 [0.99,1]	0.99 [0.99,1]
	<0.001	0.003	0.004	0.001	0.02	0.03	0.03	0.02	0.02	0.04	0.06	0.06

[a]Odds of one or more occurrences of doubt language, which refers to words or phrases that cast uncertainty upon a patient's reported clinical history (e.g., symptoms, experiences, or health behaviors). Examples include: claims, reports, denies.

[b]For all models, we included a random effect for clinicians and clustered SEs at the level of the clinician to reflect correlations in clinicians' documentation practices; hospital fixed effects to account for hospital-level variation in documentation practices.

[c]All adjusted models included adjustment for patient characteristics (age, sex, primary language, marital status, Elixhauser comorbidity index, insurance provider) and encounter characteristics (discharge location, timing of the hospital encounter before or after start of COVID-19 pandemic).

[d]We include interaction term between patient race and ethnicity and patient sex.

Abbreviations: NHW, Non-Hispanic White; NHB, Non-Hispanic Black; POC, People of Color.

© 2024 Lee CR et al. *JAMA Network Open.*

eTable 5. Unadjusted Odds Ratios of Occurrence of Evidential[a] and Epistemic Modality[b] by Race

	**Unadjusted OR[c]**
**Evidential**	1.41
(95%CI)	[1.33, 1.49]
P	<0.001
**Epistemic modality**	1.27
(95%CI)	[1.05, 1.54]
P	0.02

[a]Evidential refers to terms that indicate information was not personally obtained or observed by the writer, but that information was obtained through a secondhand source

[b]Epistemic modality refers to that indicate that validity of information is not assumed, but the level of certainty depends on the assertive word before or within the clause

[c]Comparisons are between Non-Hispanic White (NHW) patients and Non-Hispanic Black (NHB) patients only because of the relatively large prevalence of patients in these groups. For all models, we included a random effect for clinicians and clustered SEs at the level of the clinician to reflect correlations in clinicians' documentation practices; hospital fixed effects to account for hospital-level variation in documentation practices. No models included adjustment for patient or encounter characteristics. These analyses are considered post hoc secondary analysis.

© 2024 Lee CR et al. *JAMA Network Open.*

eTable 6. Subgroup Analysis of Intensive Care Unit (ICU) Documentation: Patient, Encounter, and Admission Note Characteristics by Hospital Sites

	HOSPITALS A-C Combined	HOSPITAL A	HOSPITAL B	HOSPITAL C	MIMIC-III
**N**	9277	4460	1618	3199	5649
**Age, years (mean (SD))**	64.1 (15.1)	63.0 (14.9)	63.1 (16.1)	66.2 (14.8)	63.9 (17.7)
**Sex (%)**					
Female	3671 (39.6)	1840 (41.3)	642 (39.7)	1189 (37.2)	2620 (46.4)
Male	5606 (60.4)	2620 (58.7)	976 (60.3)	2010 (62.8)	3029 (53.6)
**Race and ethnicity (%)**					
Asian/Pacific Islander	210 (2.3)	130 (2.9)	37 (2.3)	43 (1.3)	152 (2.7)
Hispanic	55 (0.6)	26 (0.6)	14 (0.9)	15 (0.5)	223 (3.9)
Non-Hispanic Black	3697 (39.9)	1432 (32.1)	532 (32.9)	1733 (54.2)	785 (13.9)
Non-Hispanic White	5066 (54.6)	2723 (61.1)	1003 (62.0)	1340 (41.9)	4376 (77.5)
Other	249 (2.7)	149 (3.3)	32 (2.0)	68 (2.1)	113 (2.0)
**Marital status (%)**					
Married/Life Partner	4557 (49.1)	2475 (55.5)	773 (47.8)	1309 (40.9)	2480 (43.9)
Not Married	4720 (50.9)	1985 (44.5)	845 (52.2)	1890 (59.1)	3169 (56.1)
**Primary language (%)**					
English	9065 (97.7)	4348 (97.5)	1577 (97.5)	3140 (98.2)	5097 (90.2)
Not English	212 (2.3)	112 (2.5)	41 (2.5)	59 (1.8)	552 (9.8)
**Insurance provider (%)**					
Medicaid	1422 (15.3)	574 (12.9)	252 (15.6)	596 (18.6)	814 (14.4)
Medicare	5553 (59.9)	2602 (58.3)	974 (60.2)	1977 (61.8)	3280 (58.1)
Employer-based[a]	2302 (24.8)	1284 (28.8)	392 (24.2)	626 (19.6)	1555 (27.5)
**Admission location (%)**					
Emergency Room	7624 (82.2)	3665 (82.2)	1172 (72.5)	2787 (87.2)	4331 (76.7)
OBGYN/Elective admission	1648 (17.8)	795 (17.8)	445 (27.5)	408 (12.8)	614 (10.9)

© 2024 Lee CR et al. *JAMA Network Open.*

Transfer from facility or outside hospital	N/A	N/A	N/A	N/A	704 (12.5)
**Discharge location (%)**					
Home/Skilled nursing facility/Rehabilitation	9146 (98.6)	4417 (99.0)	1584 (97.9)	3145 (98.3)	5574 (98.7)
Self-Discharge[b]	131 (1.4)	43 (1.0)	34 (2.1)	54 (1.7)	75 (1.3)
**Encounter length of stay, days (mean (SD))**	12.6 (18.2)	14.8 (22.3)	9.4 (12.0)	11.1 (13.4)	
**Elixhauser comorbidity index (mean (SD))**	12.2 (10.0)	13.3 (10.0)	9.5 (9.7)	12.0 (9.7)	9.2
**Timing of encounter (%)[c]**					
Before COVID-19 Pandemic	4228 (45.6)	2012 (45.1)	750 (46.4)	1466 (45.8)	5649
During or after COVID-19 Pandemic	5049 (54.4)	2448 (54.9)	868 (53.6)	1733 (54.2)	N/A
**Number of words per note (median (IQR))**	1559 (954, 2428)	1579 (971.5, 2492)	1445.5 (704, 2278)	1584 (1031, 2410)	1196 (825, 1575)
**Number of notes per clinician (median (IQR))**	2 (1, 5)	2 (1, 4)	2 (1, 4)	2 (1, 3)	8 (3, 19)

[a]Employer-based insurance provider includes employer-based, self-pay, and private insurance.

[b]Self-discharge refers to a patient who discharges against medical advice.

[c]We designated March 1, 2020 as the approximate date when clinical practice behaviors changed in response with the COVID-19 Pandemic

© 2024 Lee CR et al. *JAMA Network Open.*

eTable 7. Subgroup Analysis of Intensive Care Unit (ICU) Documentation: Number of Times Doubt Language Occurred in ICU Admission Notes by Hospital Site

	**Site**				
**Keyword**	**Hospitals A-C Combined**	**Hospital A**	**Hospital B**	**Hospital C**	**MIMIC-III**
**N**	9277	4460	1618	3199	5649
**Note with at least one word, No. (%)**	5365 (57.83)	2360 (52.91)	997 (61.62)	2008 (62.77)	3406 (60.29)
complains	650 (7.01)	264 (5.92)	110 (6.8)	276 (8.63)	3406 (60.29)
denies	2998 (32.32)	1250 (28.03)	620 (38.32)	1128 (35.26)	662 (11.72)
endorses	915 (9.86)	417 (9.35)	214 (13.23)	284 (8.88)	2473 (43.78)
notes	254 (2.74)	128 (2.87)	44 (2.72)	82 (2.56)	259 (4.58)
reports	2995 (32.28)	1334 (29.91)	612 (37.82)	1049 (32.79)	120 (2.12)
says	297 (3.2)	95 (2.13)	72 (4.45)	130 (4.06)	1505 (26.64)
states	1384 (14.92)	528 (11.84)	291 (17.99)	565 (17.66)	123 (2.18)
claims	18 (0.19)	2 (0.04)	2 (0.12)	14 (0.44)	657 (11.63)
describes	315 (3.4)	116 (2.6)	76 (4.7)	123 (3.84)	27 (0.48)
mentions	18 (0.19)	6 (0.13)	5 (0.31)	7 (0.22)	218 (3.86)
believes	34 (0.37)	15 (0.34)	8 (0.49)	11 (0.34)	13 (0.23)
according	24 (0.26)	15 (0.34)	4 (0.25)	5 (0.16)	20 (0.35)
per patient	416 (4.48)	192 (4.3)	79 (4.88)	145 (4.53)	10 (0.18)

© 2024 Lee CR et al. *JAMA Network Open.*

eTable 8. Subgroup Analysis of Intensive Care Unit (ICU) Documentation: Regression Estimates of Unadjusted and Adjusted Odds Ratios between Patient Characteristics and the Presence of Any Doubt Language in Admission Notes, by Hospital Site

	HOSPITALS A-C Combined			HOSPITAL A			HOSPITAL B			HOSPITAL C			HOSPITALS A-C Combined		
	Unadjusted OR[b] (95%CI)	Adjusted OR[b,c] (95%CI)	Adjusted OR[b,c,d] (95%CI)	Unadjusted OR[b] (95%CI)	Unadjusted OR[b] (95%CI)	Adjusted OR[b,c] (95%CI)	Adjusted OR[b,c,d] (95%CI)	Unadjusted OR[b] (95%CI)	Unadjusted OR[b] (95%CI)	Adjusted OR[b,c] (95%CI)	Adjusted OR[b,c,d] (95%CI)	Unadjusted OR[b] (95%CI)	Unadjusted OR[b] (95%CI)	Adjusted OR[b,c] (95%CI)	Adjusted OR[b,c,d] (95% CI)
**Characteristics**															
**Age**	1 [0.99,1]	1 [0.99,1]	1 [0.99,1]	1 [0.99,1]	1 [0.99,1]	1 [0.99,1]	1 [0.99,1]	1 [0.98,1.01]	1 [0.98,1.01]	0.99 [0.99,1]	1 [0.99,1.01]	1 [0.99,1.01]	1 [0.99,1]	1 [0.99,1.00]	1 [0.99,1.00]
**Female**	0.02<br>0.95 [0.86,1.06]	0.4<br>0.93 [0.84,1.04]	0.41<br>1.01 [0.87,1.18]	0.07<br>1 [0.85,1.16]	0.31<br>0.94 [0.81,1.1]	0.31<br>0.94 [0.77,1.15]	0.25<br>1.09 [0.8,1.49]	0.51<br>1.09 [0.79,1.5]	0.51<br>1.41 [0.92,2.16]	0.05<br>0.87 [0.73,1.04]	0.69<br>0.87 [0.72,1.05]	0.76<br>1.04 [0.77,1.39]	0.02<br>0.93 [0.83,1.06]	0.31<br>0.94 [0.83,1.07]	0.31<br>0.95 [0.82,1.09]
**Non-Hispanic Black**	0.38<br>1.27 [1.13,1.42]	0.21<br>1.22 [1.08,1.39]	0.86<br>1.3 [1.11,1.52]	0.95<br>1.3 [1.1,1.54]	0.46<br>1.25 [1.04,1.5]	0.56<br>1.24 [0.98,1.56]	0.59<br>0.9 [0.65,1.25]	0.61<br>0.85 [0.6,1.21]	0.11<br>1.03 [0.66,1.61]	0.13<br>1.34 [1.11,1.63]	0.14<br>1.4 [1.14,1.73]	0.82<br>1.56 [1.21,2.01]	0.28<br>1.07 [0.89,1.28]	0.38<br>1.08 [0.90,1.30]	0.47<br>1.1 [0.83,1.45]
**POC (excluding NHB)**	<0.001<br>0.87 [0.69,1.09]	0.002<br>0.85 [0.67,1.09]	0.001<br>0.98 [0.72,1.33]	0.002<br>0.97 [0.72,1.31]	0.02<br>0.94 [0.68,1.29]	0.07<br>0.97 [0.65,1.45]	0.54<br>0.7 [0.36,1.34]	0.38<br>0.54 [0.26,1.14]	0.88<br>0.87 [0.35,2.2]	0.003<br>0.8 [0.51,1.25]	0.002<br>0.87 [0.54,1.41]	0.001<br>1.06 [0.57,1.97]	0.47<br>1.06 [0.84,1.32]	0.39<br>1.2 [0.93,1.54]	0.52<br>1.21 [0.88,1.66]
	0.23	0.21	0.89	0.85	0.68	0.88	0.28	0.11	0.77	0.33	0.57	0.85	0.64	0.15	0.23
**Female * Non-Hispanic Black**			0.86 [0.69,1.07]			1.02 [0.73,1.43]			0.59 [0.31,1.16]			0.76 [0.52,1.1]			0.98 [0.68,1.41]
**Female * POC (excluding NHB)**			0.18<br>0.71 [0.44,1.13]			0.91<br>0.91 [0.5,1.69]			0.13<br>0.29 [0.08,1.14]			0.15<br>0.6 [0.24,1.51]			0.92<br>0.97 [0.62,1.54]
**Not English**	0.88 [0.62,1.24]	1.04 [0.72,1.51]	0.15<br>1.05 [0.72,1.53]	0.96 [0.59,1.57]	1.05 [0.62,1.76]	0.77<br>1.05 [0.62,1.76]	1.38 [0.52,3.66]	2.2 [0.72,6.71]	0.08<br>2.34 [0.76,7.19]	0.58 [0.31,1.09]	0.73 [0.37,1.43]	0.28<br>0.75 [0.38,1.49]	0.75 [0.61,0.92]	0.71 [0.57,0.89]	0.91<br>0.71 [0.57,0.9]
**Married/Life Partner**	0.46<br>0.9 [0.81,1.01]	0.84<br>1.01 [0.9,1.14]	0.8<br>1.01 [0.9,1.14]	0.88<br>0.84 [0.72,0.98]	0.86<br>0.93 [0.79,1.09]	0.86<br>0.93 [0.79,1.09]	0.52<br>0.98 [0.72,1.32]	0.17<br>1.05 [0.75,1.45]	0.14<br>1.03 [0.74,1.44]	0.09<br>1.01 [0.84,1.21]	0.36<br>1.11 [0.91,1.35]	0.41<br>1.11 [0.91,1.35]	0.005<br>1.03 [0.91,1.16]	0.003<br>1.05 [0.92,1.2]	0.004<br>1.06 [0.93,1.2]
	0.06	0.83	0.84	0.03	0.36	0.37	0.87	0.79	0.85	0.93	0.32	0.33	0.69	0.45	0.39

© 2024 Lee CR et al. *JAMA Network Open.*

**Medicaid (ref = Medicare)**	1.32 [1.13,1.53]	1.17 [0.97,1.4]	1.18 [0.98,1.41]	1.27 [1.01,1.6]	1.09 [0.83,1.43]	1.09 [0.83,1.43]	1.36 [0.89,2.09]	1.27 [0.75,2.15]	1.27 [0.75,2.15]	1.26 [0.99,1.59]	1.1 [0.82,1.47]	1.12 [0.83,1.5]			
	<0.001	0.1	0.08	0.04	0.56	0.55	0.16	0.38	0.38	0.06	0.53	0.46			
**private /employer-based/self-pay (ref = Medicare)**	0.97 [0.86,1.1]	0.97 [0.84,1.11]	0.97 [0.84,1.11]	0.94 [0.79,1.12]	0.92 [0.76,1.11]	0.92 [0.76,1.11]	1 [0.7,1.43]	0.96 [0.64,1.45]	0.95 [0.63,1.44]	1.07 [0.85,1.34]	1.02 [0.79,1.3]	1.03 [0.8,1.33]			
	0.67	0.64	0.64	0.49	0.38	0.38	1	0.86	0.81	0.57	0.9	0.8			
**AMA**	1.38 [0.88,2.15]	1.16 [0.73,1.82]	1.15 [0.73,1.82]	1.28 [0.6,2.72]	1.09 [0.5,2.34]	1.09 [0.51,2.34]	2.12 [0.68,6.54]	1.8 [0.56,5.82]	1.78 [0.55,5.79]	1.1 [0.56,2.14]	0.97 [0.49,1.93]	0.97 [0.49,1.92]	1.74 [1.01,3]	1.58 [0.91,2.75]	1.55 [0.89,2.69]
	0.16	0.53	0.54	0.53	0.84	0.83	0.19	0.32	0.34	0.79	0.93	0.92	0.05	0.11	0.12
**Post Covid**	0.78 [0.69,0.89]	0.76 [0.67,0.86]	0.76 [0.67,0.86]	0.77 [0.64,0.92]	0.75 [0.63,0.9]	0.75 [0.63,0.9]	0.67 [0.45,1]	0.68 [0.46,1.02]	0.69 [0.46,1.03]	0.85 [0.7,1.04]	0.82 [0.67,1]	0.82 [0.67,1.01]			
	<0.001	<0.001	<0.001	0.004	0.002	0.002	0.05	0.06	0.07	0.12	0.06	0.06			
**site B (ref = A)**	1.89 [1.52,2.35]	1.86 [1.49,2.32]	1.85 [1.48,2.31]												
	<0.001	<0.001	<0.001												
**site C (ref = A)**	1.33 [1.15,1.55]	1.25 [1.07,1.46]	1.25 [1.07,1.46]												
	<0.001	0.004	0.005												
**Elixhauser comorbidity index**	0.99 [0.99,1]	1 [0.99,1]	1 [0.99,1]	1 [0.99,1.01]	1 [0.99,1.01]	1 [0.99,1.01]	1 [0.98,1.01]	1 [0.98,1.02]	1 [0.98,1.02]	0.99 [0.98,1]	0.99 [0.98,1]	0.99 [0.98,1]	0.99 [0.98,1]	0.99 [0.98,1.00]	0.99 [0.98,1]
	0.009	0.07	0.06	0.54	0.67	0.67	0.56	0.92	0.95	0.01	0.009	0.009	0.006	0.02	0.02

[a]Odds of one or more occurrences of doubt language, which refers to words or phrases that cast uncertainty upon a patient's reported clinical history (e.g., symptoms, experiences, or health behaviors). Examples include: claims, reports, denies.

[b]For all models, we included a random effect for clinicians and clustered SEs at the level of the clinician to reflect correlations in clinicians' documentation practices; hospital fixed effects to account for hospital-level variation in documentation practices.

[c]All adjusted models included adjustment for patient characteristics (age, sex, primary language, marital status, Elixhauser comorbidity index, insurance provider) and encounter characteristics (discharge location, timing of the hospital encounter before or after start of COVID-19 pandemic).

[d]We include interaction term between patient race and ethnicity and patient sex.

© 2024 Lee CR et al. *JAMA Network Open.*

Abbreviations: NHW, Non-Hispanic White; NHB, Non-Hispanic Black; POC, People of Color.

© 2024 Lee CR et al. *JAMA Network Open.*

eFigure 5. Subgroup Analysis of Intensive Care Unit (ICU) Documentation: Unadjusted and Adjusted Associations Patient Race and Ethnicity and the Presence of Doubt Language[a] by Hospital Site

Hospital A

Race and ethnicity	Model	OR (95% CI)	P value
NHB vs NHW	Unadjusted	1.30 (1.10, 1.54)	0.002
	Adjusted	1.25 (1.04, 1.50)	0.02
POC (excluding NHB) vs NHW	Unadjusted	0.97 (0.72, 1.31)	0.85
	Adjusted	0.94 (0.68, 1.29)	0.68

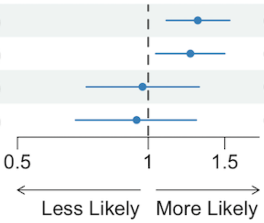

Hospital B

Race and ethnicity	Model	OR (95% CI)	P value
NHB vs NHW	Unadjusted	0.90 (0.65, 1.25)	0.54
	Adjusted	0.85 (0.60, 1.21)	0.38
POC (excluding NHB) vs NHW	Unadjusted	0.70 (0.36, 1.34)	0.28
	Adjusted	0.54 (0.26, 1.14)	0.11

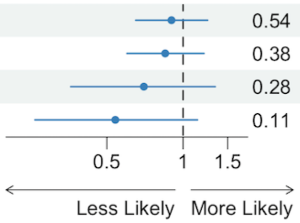

Hospital C

Race and ethnicity	Model	OR (95% CI)	P value
NHB vs NHW	Unadjusted	1.34 (1.11, 1.63)	0.003
	Adjusted	1.40 (1.14, 1.73)	0.002
POC (excluding NHB) vs NHW	Unadjusted	0.80 (0.51, 1.25)	0.33
	Adjusted	0.87 (0.54, 1.41)	0.57

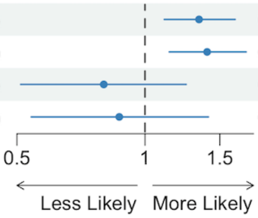

Hospitals A-C Combined

Race and ethnicity	Model	OR (95% CI)	P value
NHB vs NHW	Unadjusted	1.27 (1.13, 1.42)	<0.001
	Adjusted	1.22 (1.08, 1.39)	0.002
POC (excluding NHB) vs NHW	Unadjusted	0.87 (0.69, 1.09)	0.23
	Adjusted	0.85 (0.67, 1.09)	0.21

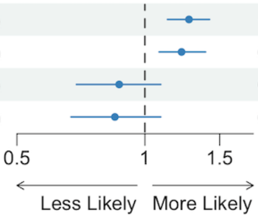

MIMIC III

Race and ethnicity	Model	OR (95% CI)	P value
NHB vs NHW	Unadjusted	1.07 (0.89, 1.28)	0.47
	Adjusted	1.08 (0.90, 1.30)	0.39
POC (excluding NHB) vs NHW	Unadjusted	1.06 (0.84, 1.32)	0.64
	Adjusted	1.20 (0.93, 1.54)	0.15

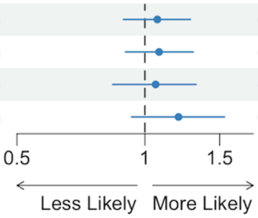

© 2024 Lee CR et al. *JAMA Network Open.*

[a]Odds of one or more occurrences of doubt language, which refers to words or phrases that cast uncertainty upon a patient's reported clinical history (e.g., symptoms, experiences, or health behaviors). Examples include: claims, reports, denies.

Comparisons are between Non-Hispanic Black (NHB) patients and Non-Hispanic White (NHW) patients and People of Color ((POC); excludes Non-Hispanic Black patients and includes Asian, Native Hawaiian or Pacific Islander, American Indian, Alaskan Native, Middle Eastern, Portuguese, or multi-racial/ethnic patients) and NHW patients. For all models, we included a random effect for clinicians and clustered SEs at the level of the clinician to reflect correlations in clinicians' documentation practices; hospital fixed effects to account for hospital-level variation in documentation practices. All adjusted models included adjustment for patient characteristics (age, sex, primary language, marital status, Elixhauser comorbidity index, insurance provider) and encounter characteristics (discharge location, timing of the hospital encounter before or after start of COVID-19 pandemic).

Abbreviations: NHW, Non-Hispanic White; NHB, Non-Hispanic Black; POC, People of Color.

© 2024 Lee CR et al. *JAMA Network Open.*

eTable 9. Unadjusted and Adjusted Regression Estimates of Patient Characteristics and Rate Ratios of Doubt Language[a]

	**Bivariate Analysis[b]**	**Multivariable Analysis[b]**
**age**	1.002	1.004
(95%CI)	[1.001,1.003]	[1.003,1.006]
P	0.004	0.000
**Female**	0.883	0.867
(95%CI)	[0.848,0.919]	[0.840,0.895]
P	0.000	0.000
**Non Hispanic Black**	1.249	1.173
(95%CI)	[1.206,1.293]	[1.135,1.212]
P	0.000	0.000
**POC**	1.011	1.050
(95%CI)	[0.960,1.066]	[0.996,1.108]
P	0.670	0.069
**Not English**	0.858	0.859
(95%CI)	[0.791,0.931]	[0.785,0.941]
P	0.000	0.001
**Married/Life Partner**		0.842
(95%CI)	[0.752,0.798]	[0.818,0.866]
P	0.000	0.000
**Medicaid**	1.131	1.148
(95%CI)	[1.085,1.180]	[1.107,1.190]
P	0.000	0.000
**Private/commercial/ employer-based/self-pay**	0.824	0.951
(95%CI)	[0.783,0.868]	[0.917,0.987]
P	0.000	0.008
**AMA**	1.451	1.294
(95%CI)	[1.346,1.564]	[1.199,1.397]
P	0.000	0.000

© 2024 Lee CR et al. *JAMA Network Open.*

**post_covid=1**	0.949	0.925
(95%CI)	[0.900,1.002]	[0.880,0.973]
P	0.057	0.003
**Hosp B**	1.248	1.265
(95%CI)	[1.133,1.376]	[1.156,1.385]
P	0.000	0.000
**Hosp C**	1.195	1.068
(95%CI)	[1.112,1.283]	[0.993,1.148]
P	0.000	0.075
**Elixhauser_vanwalraven**	0.999	0.996
(95%CI)	[0.997,1.001]	[0.994,0.998]
P	0.316	0.000

[a]Doubt language refers to words or phrases that cast uncertainty upon a patient's reported clinical history (e.g., symptoms, experiences, or health behaviors).

[b] [a]We selected Poission regression to evaluate the relationship between patient characteristics and the rate ratio of occurrences of any doubt language in a note. Models included random effect for clinicians standard errors were at the level of the clinician to account for clustering of admission notes and patterns in documentation within clinicians.

© 2024 Lee CR et al. *JAMA Network Open.*

eTable 10. Falsification Test: Unadjusted and Adjusted Regression Estimates of Patient Characteristics and Rate Ratios of Stop Words

	**Bivariate Analyses[a]**	**Multivariable Analysis[a]**
**age**	1.001	1.001
(95%CI)	[1.001,1.001]	[1.001,1.001]
P	0	0
**Female**	0.986	0.995
(95%CI)	[0.981,0.991]	[0.992,0.998]
P	0	0.002
**Non-Hispanic Black**	1.008	1.009
(95%CI)	[1.005,1.011]	[1.006,1.012]
P	0	0
**POC**	1.001	1.007
(95%CI)	[0.996,1.007]	[1.001,1.012]
P	0.631	0.018
**Not English**	1.004	0.999
(95%CI)	[0.995,1.014]	[0.990,1.009]
P	0.367	0.906
**Married/Life Partner**	0.986	0.986
(95%CI)	[0.983,0.989]	[0.983,0.988]
P	0	0
**AMA**	1.033	1.035
(95%CI)	[1.025,1.041]	[1.027,1.043]
P	0	0
**post_covid=1**	1.002	1
(95%CI)	[0.997,1.007]	[0.995,1.004]
P	0.442	0.855
**Hosp B**	0.959	0.961
(95%CI)	[0.950,0.967]	[0.954,0.968]
P	0	0
**Hosp C**	0.993	0.982
(95%CI)	[0.988,0.999]	[0.977,0.987]

© 2024 Lee CR et al. *JAMA Network Open.*

P	0.014	0

[a]We selected Poission regression to evaluate the relationship between patient race and ethnicity and use of stop words as a falsification test because any stop word likely appears at least once in an admission note. Models included random effect for clinicians standard errors were at the level of the clinician to account for clustering of admission notes and patterns in documentation within clinicians. Example of stop words include: a, an, the.

© 2024 Lee CR et al. *JAMA Network Open.*
