# Supplementary material for: Race and Ethnicity and Clinician Linguistic Expressions of Doubt in Hospital Admission Notes
Source: JAMA Netw Open. 2024 Oct 14;7(10):e2438550. doi: 10.1001/jamanetworkopen.2024.38550 (PMC11581534; doi:10.1001/jamanetworkopen.2024.38550)
Supplement: Supplement 2. — Data Sharing Statement [file jamanetwopen-e2438550-s002.pdf]

Data Sharing Statement

Lee. Race and Ethnicity and Clinician Linguistic Expressions of Doubt in Hospital Admission Notes. *JAMA Netw Open*. Published online October 14, 2024. doi:10.1001/jamanetworkopen.2024.38550

Data

Data available: Yes
Data types: Data dictionary
How to access data: Data dictionary will be available upon request to email - courtneyrlee64@gmail.com
When available: With publication

Supporting Documents

Document types: Statistical/analytic code
How to access documents: Statistical/analytic code will be made available upon request to courtneyrlee64@gmail.com
When available: With publication

Additional Information

Who can access the data: The data dictionary will be made available to anyone requesting the information
Types of analyses: Once approved, the data dictionary will be made available for a specified purpose.
Mechanisms of data availability: The data dictionary will be made available with approval of a proposal.
